# ASPH-notch Axis guided Exosomal delivery of Prometastatic Secretome renders breast Cancer multi-organ metastasis

**DOI:** 10.1186/s12943-019-1077-0

**Published:** 2019-11-07

**Authors:** Qiushi Lin, Xuesong Chen, Fanzheng Meng, Kosuke Ogawa, Min Li, Ruipeng Song, Shugeng Zhang, Ziran Zhang, Xianglu Kong, Qinggang Xu, Fuliang He, Xuewei Bai, Bei Sun, Mien-Chie Hung, Lianxin Liu, Jack Wands, Xiaoqun Dong

**Affiliations:** 1 Department of Internal Medicine, College of Medicine, The University of Oklahoma Health Sciences Center, Oklahoma City, OK 731014 USA; 2 Department of Internal Medical Oncology, Harbin Medical University Cancer Hospital, Harbin, 150040 Heilongjiang Province People’s Republic of China; 3 Department of Hepatic Surgery, Key Laboratory of Hepatosplenic Surgery, Ministry of Education, The First Affiliated Hospital of Harbin Medical University, Harbin, China; 4 Liver Research Center, Rhode Island Hospital, Warren Alpert Medical School, Brown University, 55 Claverick Street, 4th Fl., Providence, RI 02903 USA; 5 Immunobiology & Transplant Science Center, Houston Methodist Research Institute, Houston, TX 77030 USA; 6 Institute of Life Sciences, Jiangsu University, No. 301 Xuefu Road, Zhenjiang, 212013 Jiangsu Province, People’s Republic of China; 7 Department of Interventional Therapy, Beijing Shijitan Hospital, Capital Medical University, The 9th affiliated hospital of Peking University, Beijing, People’s Republic of China; 8 Department of Pancreatic and Biliary Surgery, The First Affiliated Hospital of Harbin Medical University, 23 Youzheng Street, Nangang District, Harbin, 150001 Heilongjiang Province People’s Republic of China; 9 Department of Molecular and Cellular Oncology, The University of Texas MD Anderson Cancer Center, Houston, TX 77030 USA; 10 Graduate School of Biomedical Science, The University of Texas Health Science Center at Houston, Houston, TX 77030 USA; 11 Division of Life Sciences and Medicine, The First Affiliated Hospital of USTC, The University of Sciences and Technology of China, No. 17 Lujiang Road, Hefei City, 230001 An Hui Province People’s Republic of China; 12 Division of Gastroenterology, Department of Medicine, The Warren Alpert Medical School, Brown University, Providence, RI 02903 USA

**Keywords:** Aspartate β-hydroxylase (ASPH), Exosome, Notch, Metastasis, Breast cancer

## Abstract

**Background:**

Aspartate β-hydroxylase (ASPH) is silent in normal adult tissues only to re-emerge during oncogenesis where its function is required for generation and maintenance of malignant phenotypes. Exosomes enable prooncogenic secretome delivering and trafficking for long-distance cell-to-cell communication. This study aims to explore molecular mechanisms underlying how ASPH network regulates designated exosomes to program development and progression of breast cancer.

**Methods:**

Stable cell lines overexpressing or knocking-out of ASPH were established using lentivirus transfection or CRISPR-CAS9 systems. Western blot, MTT, immunofluorescence, luciferase reporter, co-immunoprecipitation, 2D/3-D invasion, tube formation, mammosphere formation, immunohistochemistry and newly developed in vitro metastasis were applied.

**Results:**

Through physical interactions with Notch receptors, ligands (JAGs) and regulators (ADAM10/17), ASPH activates Notch cascade to provide raw materials (especially MMPs/ADAMs) for synthesis/release of pro-metastatic exosomes. Exosomes orchestrate EMT, 2-D/3-D invasion, stemness, angiogenesis, and premetastatic niche formation. Small molecule inhibitors (SMIs) of ASPH’s β-hydroxylase specifically/efficiently abrogated in vitro metastasis, which mimics basement membrane invasion at primary site, intravasation/extravasation (transendothelial migration), and colonization/outgrowth at distant sites. Multiple organ-metastases in orthotopic and tail vein injection murine models were substantially blocked by a specific SMI. ASPH is silenced in normal adult breast, upregulated from in situ malignancies to highly expressed in invasive/advanced ductal carcinoma. Moderate-high expression of ASPH confers more aggressive molecular subtypes (TNBC or Her2 amplified), early recurrence/progression and devastating outcome (reduced overall/disease-free survival) of breast cancer. Expression profiling of Notch signaling components positively correlates with ASPH expression in breast cancer patients, confirming that ASPH-Notch axis acts functionally in breast tumorigenesis.

**Conclusions:**

ASPH-Notch axis guides particularly selective exosomes to potentiate multifaceted metastasis. ASPH’s pro-oncogenic/pro-metastatic properties are essential for breast cancer development/progression, revealing a potential target for therapy.

## Background

Breast cancer is the most common malignancy (accounting for 25% of all patients) in women, with 1.7 million new cases diagnosed worldwide in 2012. In USA, it is the 2nd leading cause of cancer-related death, with an estimated 266,120 new cases and 40,920 deaths in 2018 [1]. For patients with aggressive tumors that are refractory to multiple treatments, a dismal prognosis is expected and a vast majority die of widespread diseases. The purpose of this study is to evaluate potential value of aspartate β-hydroxylase (ASPH) as a therapeutic target for breast cancer metastasis. ASPH has natural substrates, i.e., aspartyl and asparaginyl residues in EGF-like repeats of various proteins, in particular Notch receptors and ligands [2–8]. Notch pathway is essential for differentiation, cell fate determination, adhesion, proliferation, migration, invasion and stemness [9]. ASPH is silent in normal adult tissues, however, during tumorigenesis, it is re-emerged and markedly upregulated by growth factor signaling pathways IN/IGF1/IRS1/RAS/RAF/MAPK/ERK, IN/IGF1/IRS1/PI3K/AKT, and WNT/β-catenin [8, 10–13] attributed to development and progression of malignancies [14–16]. ASPH protein is overexpressed in a variety of malignancies, such as non-small cell lung cancer (NSCLC), pancreatic cancer, cholangiocarcinoma [15], hepatocellular carcinoma (HCC) and colorectal cancer [14, 15, 17, 18]. Notably, high expression levels of ASPH and its truncated homolog Humbug predict reduced survival of patients with NSCLC [19], HCC^16^ and colon cancer [20]. Upregulated ASPH augments cell migration and invasion [21]. However, it is yet to be illustrated whether Notch cascade activated by ASPH is a major molecular mechanism to generate and maintain malignant cellular behaviors in breast cancer [8].

Exosomes (30–100 nm) are multi-purpose carriers of a broad spectrum of biologically active cargoes. As key mediators for communication, exosomes deliver various molecules with specific properties to exchange complex information during tumor development, progression [22] and metastasis [23]. Cellular components of tumor-microenvironment secrete exosomes in an autocrine/a paracrine fashion to promote tumor-induced immunosuppression, angiogenesis, and pre-metastatic niche formation [24, 25]. In this study, we attempt to explore whether: (a) ASPH-Notch axis is involved in exosomal cell-to-cell communication; (b) ASPH-Notch signaling biologically depends on exosomal delivery and functions to initiate breast cancer progression; (c) inhibition of ASPH-Notch axis can reverse aggressive phenotypes and consequently block breast cancer pathogenesis?

## Methods

### Cell lines

The human breast cancer cell lines MDA-MB-231, MDA-MB-468, T47D, Au565, SKBR3, and MCF-7; normal breast cell lines MCF-10A and MCF-12A were kind gifts from Dr. Mien Chie Hung at The University of Texas MD Anderson Cancer Center. HEK293, 293 T, HCC1937, BT-549, BT-474 and HUVEC (human umbilical vein endothelial cells) were purchased from American Type Culture Collection (ATCC). HUVECs grown in complete F-12 K medium were used at passages 5–10. All cell lines were authenticated by short tandem repeat profiling to reduce the frequency of misidentification. Cells were cultured at 37 °C in a humidified atmosphere containing 5% CO_2_ in a corresponding medium supplemented with 10% FBS and antibiotics (penicillin and streptomycin). Cells were passaged when reached 80% confluence. Stable MDA-MB-231 and MDA-MB-468 expressing empty vector, wild-type (WT)-ASPH, and H675Q mutant ASPH were established using lentiviral system (GeneCopoeia, #EX-Z8758-Lv105), whereas stable T47D and BT-474 expressing CRISPR vector and ASPH knockout using CRISPR-CAS9 system. All stable cell lines stably expressing GFP were generated using lentiviral system for in vitro metastasis assays.

### Plasmids and reagents

Plenti-CMV-ASPH-Lv105 (EX-Z8758-Lv105) and Plenti-CMV- Lv105 empty vector, pReceiver-CMV-CD63 eGFP, pReceiver-CMV-MMP2 mCherry and pReceiver-CMV-JAG1(EX-M0722-M02) plasmids were purchased from GeneCopoeia; Notch1 reporter construct (12XCSL-DsRe-dExpressDL, 47,683), pcDNA3-ADAM17-HA (#65105), pLenti-CMV-GFP-Hygro (656–4) and lentiCRISPR v2 from Addgene. The pcDNA3 NOTCH1 full-length plasmid construct containing codons 1 to 2555 of NOTCH1 followed by a FLAG tag sequence was a kind gift from Dr. Iannis Aifantis at NYU School of Medicine.

DAPT (D5942-5MG; γ-secretase inhibitor), GW4869 (567715-1MG; N-SMase inhibitor) and GM6001 (CC1010; pan-MMPs inhibitor) were purchased from Sigma-Aldrich and examined at multiple concentrations within effective range and with minimal off-target effects or toxicity.

### Western blot

Cell lysates (20–40 μg) were separated by SDS-PAGE and transferred to nitrocellulose membranes. Primary antibodies against the following molecules were applied: ASPH (homemade); JAG1 (70109), JAG2 (2205), DLL1 (2588), DLL4 (2589), Notch1 (3608), Notch2 (4530), Notch3 (5276), Notch4 (2423), c-Myc (13987), MMP2 (87809), MMP7 (3801), ADAM9 (2099), ADAM10 (14194), ADAM17 (3976), EpCAM (2929 and 5198) and CD44 (5640) from Cell Signaling Technology; activated Notch1 (Abcam ab8925), HES1 (ab71559) and HES2 (ab134685) from Abcam; MMP9 (sc-13,520), CD63 (sc-15,363) from Santa Cruz Biotechnology; and HEY1 (AB5714) from Millipore. Protein bands were visualized with ChemiDoc™ Touch Imaging System (Biorad).

### Immunoprecipitation

Cells were cultured in 10-cm dishes, transfected with 10 μg corresponding plasmids when reached 70% confluence, and harvested using 1 ml PBS containing 1% NP40.

### Luciferase reporter

Cells were seeded in 24-well plates at 50–60% confluence, incubated for 24 h, and transfected with 100 ng of Notch1 ± 100 ng ASPH, 50 ng Notch1 (full-length; FL), or 25 ng Active Notch1 (NICD) plasmids/well in serum-free medium. Total amounts of these plasmids were balanced with an empty pcDNA3 plasmid. After 8 h of transfection, medium was replaced with normal medium ± DMSO, or MO-I-1182 (the 3rd generation SMI of ASPH enzymatic activity) [2, 11] at 50 nM, or DAPT at 10 μM, respectively; and incubated for 40 h. Cells were imaged by fluorescence microscope. All experiments were performed in triplicate wells for each condition and repeated in triplicate.

### Tube formation

300 μl of Matrigel Matrix (10 mg/ml) was added to each well of a 24-well plate on ice. The plate was centrifuged at 4 °C, 300×g for 10 min, and immediately incubated at 37 °C for 60 min. 300 μl of HUVEC suspension (1.2 × 10^5^) ± exosomes (5 μg/ml) was added to each well. The plate was incubated for 12-24 h at 37 °C, in 5% CO_2_. Tube-like structure was imaged by microscope. The number of vessel joints was counted. Tube area was calculated by NIH Image J. Results from three independent experiments represented as mean ± SD.

### Mammosphere formation

300 μl of Matrigel was spread evenly to each well of a 24-well plate on ice. The plate was centrifuged at 4 °C, 300×g; and immediately incubated at 37 °C, in 5% CO_2_ for 30 min. Single-cells were suspended in corresponding medium with 10% Matrigel (2000 cells/400 μl) and seeded on Matrigel. Cells were allowed to attach to Matrigel for 3 h. Then medium was carefully removed, replaced with fresh one containing 10% Matrigel and incubated for 1 h. Fresh medium containing Matrigel was changed every 2 days. Mammospheres formed after 5–9 days were evaluated in terms of size and number by light microscopy. All experiments were performed in triplicate wells for each condition and repeated in triplicate.

### 3D-embedded cultures (cancer cell lines embedded); 3D-on top cultures (coculture layer epi−/endothelial cells on top)

24-well plate was coated with 300 μl/well of growth factor reduced (GFR) Matrigel and incubated at 37 °C in 5% CO_2_ for 30 min. Cells were harvested, counted and diluted to a concentration of 5000 cells/ml in complete growth medium (containing 2% GFR Matrigel). 400 μl/well of cell suspension was added to Matrigel pre-coated plate and incubated in 5% CO_2_ at 37 °C for at least 4 h. Cells were observed every day and images were obtained at 5–8 days. To evaluate potential effects of different pharmacologic inhibitors, compounds were added to complete media at the time of seeding, followed by changing fresh media supplemented with compounds every day when the cells growing on GFR Matrigel. All experiments were performed in triplicate wells for each condition and repeated in triplicate.

### 3-D (spheroid) invasion

3-D Culture 96-Well BME Cell Invasion (Trevigen Inc. Gaithersburg, MD) more comprehensively and physiologically mimics in vivo scenario. Cell monolayers were washed with PBS, dissociated by Trypsin and neutralized with complete growth medium. Cell suspension was counted by a hemocytometer and diluted to 1 × 10 [4]/ml, to obtain spheroids of 300–500 μm in diameter at ≥4 days after seeded. The cell suspension was dispensed into ULA 96-well round bottom plate and centrifuged at 200×g for 5 min. The plate was transferred to an incubator (37 °C, 5% CO_2_, 95% humidity). After 3–5 days, tumor spheroid formation was visually confirmed and proceeded with 3-D invasion. Basement membrane matrix (BMM) was thawed on ice overnight. ULA 96-well plate containing 4-day old spheroids was placed on ice. 50 μl of BMM was gently dispensed into each U-bottom well with 6 replicates for each condition. The plate was centrifuged at 4 °C, 300×g for 3 min, then transferred to an incubator at 37 °C, allowing the BMM to solidify. After 1 h, 100 μl/well of complete growth medium was gently added. Invasion modulating agent (SMI, DAPT, GW4869 or GM6001) was applied to evaluate respective impact on cellular phenotype. Cell invasion was visualized microscopically and quantitated with NIH IMAGEJ.

### ECM degradation/remodeling

Cover glass (18 mm; Fisher Scientific) was coated with pig skin green 488 conjugated Gelatin (G13186, Life Technologies). The gelatin was cross-linked with a 0.5% glutaraldehyde solution in a 12-well plate, followed by quenched with sodium borohydride (1 mg/ml) and washed 3 times with PBS. Breast cancer cells (2 × 10 [4]) were seeded to each well in 2 ml of complete medium. After 18-72 h, cells were fixed with 4% paraformaldehyde (PFA), permeabilized with 0.1% Triton X-100, blocked with 5% bovine serum albumin (BSA), and probed for F-actin (Rhodamine phalloidin, R415, Life technologies). The coverslip was mounted over a glass slide with a drop of mounting medium containing DAPI (4′,6-diamidino-2-phenylindole, dihydrochloride). At least 15 fields per coverslip were imaged at all three channels (“red”, “green” and “blue”) under 400× magnification. Black and white images of gelatin degradation were analyzed using NIH IMAGEJ software. The degraded area was normalized to the number of nuclei from the same field. All experiments were performed in triplicate wells for each condition and repeated in triplicate.

### Exosome collection

Breast cancer cells were cultured in 15-cm dishes. When reached 80% confluence, cells were rinsed with PBS twice and incubated with fresh corresponding medium for 48 h. Conditioned medium was collected and the final cell number was counted by a hemocytometer. Exosomes were isolated from conditioned medium using sequential centrifugation at 4 °C: at 2000×g for 15 min to remove cell debris, and at 100,000×g for 3 h to pellet exosomes. The pellet was re-suspended in PBS and recentrifuged at 100,000×g for 3 h to obtain purified exosomes. The purified exosome pellet was resuspended in PBS and adjusted according to the final cell number to yield an equivalent secreted exosome concentration on a per cell basis. Exosomes were counted by NanoSight nanoparticle tracking analysis (NanoSight, Ltd., Amesbury, UK).

Donors released exosomes were internalization of by recipients. Exosomes were labeled and purified using Exo-Green protein fluorescent labeling kit (SBI, EXOG200A-1). Breast cancer cells were seeded at a density of 1 × 10 [5]/well in 6-well plates. 100 μl of labeled exosomes suspension was added to cells and allowed to be taken up for 24 h. The cells were washed three times with PBS, fixed with 4% formaldehyde, permeabilized with 0.1% Triton X-100, and stained with Phalloidin. Totally 50 μl of CD9-, CD63-, CD81-, and MMP2-coupled magnetic beads from Exo-Flow kit (SBI, EXOFLOW32A-CD9, −CD63, −CD81, and -MMP2) were incubated with 100 μl of labeled exosomes overnight at 4 °C. The beads were placed on magnetic plates, washed three times with wash buffer, loaded to magnetic rack, and imaged by fluorescence microscope.

### In vitro metastasis

Matrigel invasion chambers (BD BioCoat Matrigel Invasion 24-well Chamber, 8 μm pores, BD Biosciences) were rehydrated for 2 h at 37 °C with serum-free medium. HUVECs (2 × 10 [5]) in HUVEC Medium were seeded in inserted chambers. After 24 h, lower chambers were coated with 290 μl of matrigel and filled with 500 μl of HUVEC Medium containing 10% FBS. Breast cancer cells (1–4 × 10 [4]) stably expressing GFP in HUVEC Medium (FBS-free) were plated onto a layer of HUVECs. The plate was incubated in CO_2_ incubator for 3 days. Inserted chambers of 24-well plates were removed from the plates, washed with PBS, fixed with 4% PFA (Sigma-Aldrich) for 20 min, permeabilized with Triton X-100 for 20 min, and then stained with phalloidin (red) and Hoechst. Transendothelial-migrated breast cancer cells that had passed through HUVECs were imaged and counted using fluorescence microscope. Breast cancer cells that had transmigrated into the matrigel of lower chambers were buried with corresponding growth medium containing 10% matrigel, and continuously cultured for 7 days to allow mammosphere formation. Tumor spheres were imaged and measured in terms of size and number with fluorescence microscope.

### Orthotopic breast Cancer models

Animal studies were approved by the IACUC of Rhode Island Hospital, Brown University. NSG (NOD.Cg-Prkdc^scid^ Il2rg^tm1Wjl^/SzJ) mice were purchased from Jackson Laboratory. 6–8-week old female mice were injected subcutaneously with 1 × 10^6^ MDA-MB-231 cells stably expressing Vector or ASPH in 100 μL of ice-cold 50:50 Matrigel/Collagen I solution (Growth Factor Reduced and Phenol Red-free Matrigel, BD Biosciences) into the right fourth abdominal fat pad at the base of the nipple. Tumor growth was monitored externally using Vernier calipers for up to 4 weeks by measuring length (L), width (W) and depth (D). Total tumor volume was calculated using the formula: V = LWD × π/6.

The animals were euthanized with an overdose of CO_2_ exposure when developed ascites or an orthotopic tumor reaching 1 cm in diameter (or 10% of body weight). The time point of euthanasia was recorded as mortality. All surviving mice were euthanized after 4 weeks and evaluated macroscopically for orthotopic tumors and macro-metastases in potentially involved organs, such as abdominal cavity. Necropsies were performed to identify micro−/macro-metastases. Primary tumors and involved organs were harvested, weighed, fixed with 10% neutral buffered formalin, paraffin-embedded, and sectioned.

Metastatic dissemination in tumor-bearing mice was quantified by both visually (in extracted lungs using magnifying lens) and histologically (using tissue sections and light microscopy). Slides were independently analyzed by two pathologists to evaluate metastases. RNA was isolated from three independent metastatic lesions and primary tumors. Histologic characteristics were detailed from tumor tissue sections stained with hematoxylin and eosin (H&E). The microscopic images were analyzed with NIH ImageJ to distinguish tumor area from adjacent non-tumor area. To decipher the expression profiling of ASPH network components, immunohistochemistry (IHC) was conducted on 4-μm thick formalin-fixed paraffin-embedded (FFPE) sections of orthotopic tumor specimens using primary antibodies against ASPH, activated Notch1, ADAM17, and MMP2/9.

To determine anti-tumor effects of the 3rd generation SMI (MO-I-1182) as described previously [2, 11], the mice were randomly divided into 2 groups (*n* = 6/group) and treated for an established tumor (100 mm^3^) with DMSO (control) vs. SMI at a dose of 10 mg/kg, intraperitoneal (i.p.), every other day.

### Experimental lung metastatic (tail vein injection) models

Female BALB/c athymic nude mice (4–6 weeks old) were purchased from Laboratory Animal Center of Shanghai Institute for Biological Sciences (SIBS). The animals were housed under standard conditions and cared for according to the institutional guidelines. Animal experiments were approved by the IACUC of Harbin Medical University.

MDA-MB-231 cells stably expressing Vector or ASPH were detached with nonenzymic dissociation buffer, resuspended in Dulbecco’s PBS. 1 × 10^6^ cells per 200 μl PBS were immediately injected into the tail vein. Mice were treated with MO-I-1182 (10 mg/kg, every other day) or vehicle (5% DMSO) by i.p. injection starting 1 day after i.p. injection of breast cancer cells. Tumors were imaged by bioluminescence to evaluate development, progression and metastasis. D-luciferin (Xenogen, Hopkinton, MA) was injected intraperitoneally at 100 mg/kg (mouse weight). Bioluminescence was detected with Berthold NIGHTOWL LB983 imaging machine. Each mouse was photographed; and luminescent images were acquired with various periods of exposure time (1–60 s). The resulting grayscale photographic and pseudo-colored luminescent images were automatically superimposed by Living Image software to match the observed luciferase signal with its location on individual mouse. Vital status was recorded daily. After 4 weeks, the mice were sacrificed; their lungs and other involved organs when appropriate were dissected, perfused with PBS, and fixed with freshly prepared formaldehyde (4% in PBS) for H&E and IHC staining. Pulmonary micro−/macro-metastases (e.g., tumor nodules) were monitored and quantified using noninvasive bioluminescence system or by visually counting under dissection microscope. Expression profiling of ASPH-Notch network components including ASPH, activated Notch1, ADAM17, and MMP2/9 were semi-quantified by IHC.

### Patient selection and immunohistochemistry (IHC)

Tissue specimens from 141 archived de-identified individuals with breast cancer were randomly selected from tissue bank. A written informed consent was waivered. This retrospective study was approved by the Institutional Review Board of respective institution including Lifespan Rhode Island and The Miriam Hospital (USA) and The First Affiliated Hospital of Harbin Medical University (P.R. China). All procedures were conducted according to regulations and guidelines approved by ethics committee at respective institution.

Immunohistochemical staining was conducted on 4-μm FFPE unstained sections using antibodies against ASPH (homemade, FB50, 1:10000), Active Notch1 (Abcam, ab8925, 1:1000), MMP2 (Abcam, ab110186, 1:1000), MMP9 (Abcam, ab38906, 1:1000), and ADAM17/TACE (Abcam, ab39162, 1:1000). The sections were deparaffinized in xylene and rehydrated in a descending ethanol gradient. Antigen retrieval was performed using Citric Acid Based Antigen Unmasking Solution (Vector Laboratories, Burlingame, CA) in microwave pressure cooker (Nordic Ware, Minneapolis, MN) at full power for 6 min, followed by 30-min cool-down. Endogenous peroxidase activity was quenched by treatment with 3% hydrogen peroxide in methanol for 30-min. Subsequent blocking, secondary antibody incubation and ABC reagent incubation were performed using VECTASTAIN Elite ABC Kit (Vector Laboratories, Burlingame, CA) according to the manufacturer’s protocol. Primary antibodies were diluted in TBS/0.1% Tween-20 with 5% normal goat serum and incubated at 4 °C overnight. Color development was performed using DAB Tablet (Wako Chemicals, Richmond, VA) as a substrate per manufacturer’s instruction. Finally, the sections were counterstained by hematoxylin, dehydrated in an ascending ethanol gradient, and mounted with VECTASHIELD Mounting Medium (Vector Laboratories, Burlingame, CA).

### Statistical analysis

Statistical analyses were performed with SPSS (version 25) and GraphPad software packages. Nonparametric data were analyzed with Kruskal-Wallis one-way ANOVA, followed by Tamhane’s post hoc test. Data with normal distributions were represented by mean ± SD and analyzed using one-way ANOVA followed by Bonferroni post hoc. Spearman’s rank correlation coefficient (ρ) and Pearson’s correlation coefficient (r) were employed to evaluate the relationship of ASPH expression with levels of other components in breast cancer tumor tissue as calculated using IHC scoring system. The progression-free survival (PFS) was calculated from the date of surgery to the date of clinically detectable relapse, progression or metastasis, death or last follow-up. The overall survival (OS) time was calculated from the date of diagnosis to the date of death or last follow-up. Median survival time was estimated using Kaplan-Meier plot. Difference in median survival time was examined with log-rank test. Associations between ASPH expression levels with molecular subtypes and tumor characteristics (recurrence/progression and metastasis) were examined with χ^2^ or Fisher’s Exact test. A *p* value of < 0.05 (2-sided) was considered statistically significant.

## Results

### ASPH physically interacts with notch receptors, ligands, and regulators to activate notch cascade in breast cancer

Expression profile of ASPH-Notch axis was demonstrated in a panel of human breast cancer cell lines (Additional file 1: Figure S1A-B). ASPH was relatively highly expressed in T47D, BT474 and HCC1937; moderately in BT549, SKBR3 and MCF7; whereas lowly in Au565, MDA-MB-231 and MDA-MB468. Thus, MDA-MB-231 and MDA-MB-468 stably expressing empty vector vs. ASPH using lentivirus expression system; whereas T47D and BT474 stably expressing CRISPR-vector vs. ASPH knockout (KO) using CRISPR-CAS9 system (Additional file 1: Figure S1C-D) were established to explore molecular mechanisms.

**Fig. 1:**
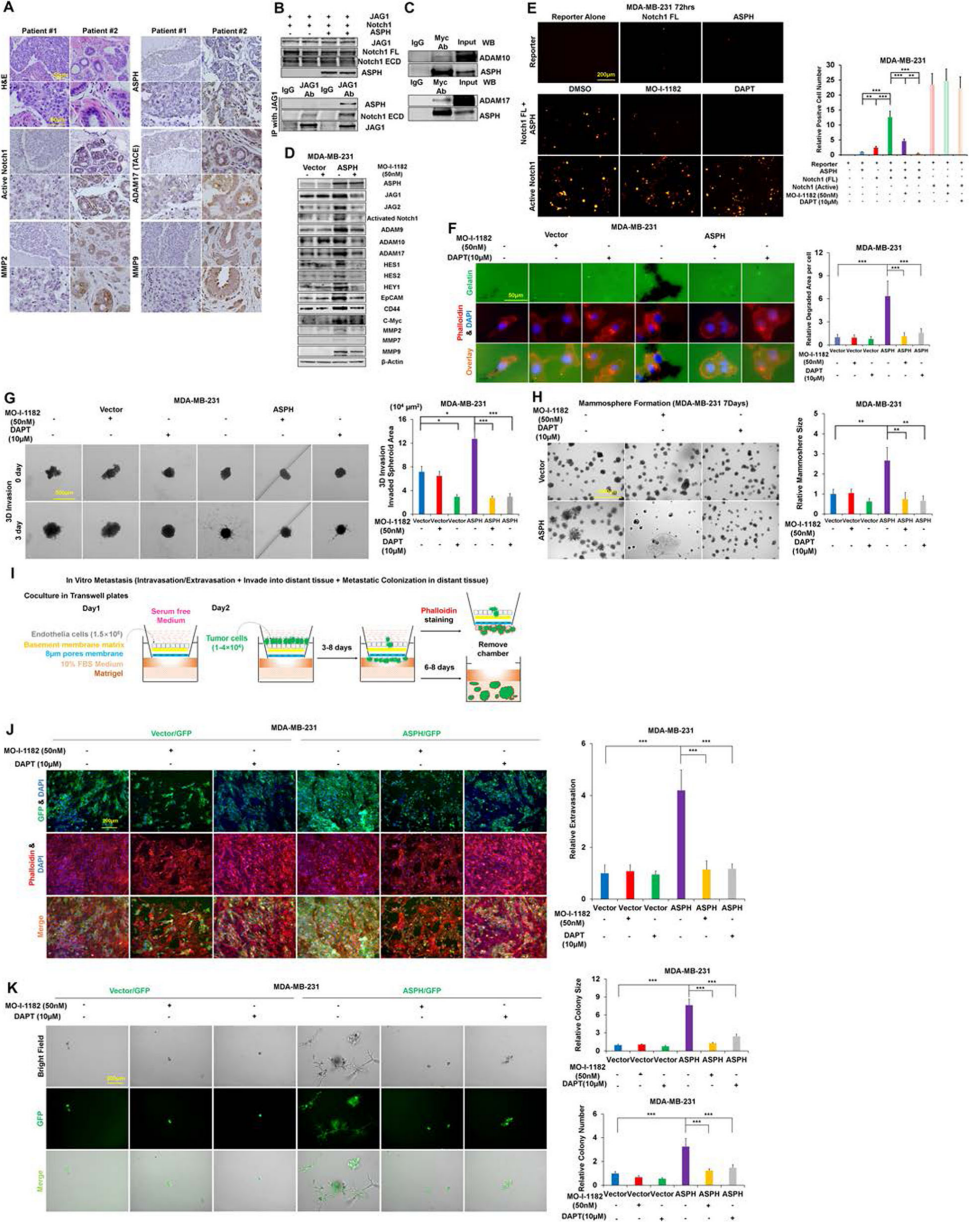
ASPH activates Notch signaling pathway in breast cancer. (**a**) First Row Left: Histopathology of 2 representative tumors derived from breast cancer patients; First Row Right: Patient #1 and #2 had ASPH negative vs. positive tumor, respectively, by IHC. Second to Fourth Row: Consistent downregulation vs. upregulation of Activated Notch1, MMP-2, MMP-9, and ADAM17/TACE in ASPH negative vs. positive breast cancer patients. (**b**-**c**) ASPH activates Notch signaling pathways through physical interaction with Notch receptors, ligands and regulators in breast cancer. ASPH physically interacts with Notch1 extracellular domain (ECD), JAG1, ADAM10 and ADAM17 as demonstrated by co-IP. ASPH enhances physical interaction between Notch1 and JAG1. (**d**) Expression profiling of Notch signaling core components in response to SMI. (**e**) Luciferase reporter to detect the activation of Notch signaling in breast cancer, in the presence of full-length (FL) Notch1 and/or ASPH. Reporter alone served as a negative control whereas active Notch1 (Notch intracellular domain; NICD) alone as a positive control. ASPH activates Notch signaling in presence of FL Notch1, which was inhibited by SMI and DAPT. (**f**) ECM degradation/remodeling in response to SMI and DAPT, respectively. (**g**) 3-D invasion in response to SMI and DAPT, respectively. (**h**) Mammosphere formation in response to SMI and DAPT, respectively. (**i**) Scheme of in vitro metastasis assay. (**j**) Transendothelial migration (intravasation/extravasation); (**k**) Invasion through basement membrane and subsequent mammosphere formation in response to SMI and DAPT, respectively. ^*^p < 0.05; ^**^p < 0.01; ^***^p < 0.001

ASPH catalyzes hydroxylation of aspartyl/asparaginyl residues in EGF-like repeats of Notch receptors and ligands. Indeed, ASPH activates Notch signaling in breast cancer patients. ASPH was highly expressed in more aggressively poorly differentiated tumors, whereas negatively-lowly expressed in less invasive moderately-well differentiated tumors (Fig. 1a; Additional file 1: Figure S1E). Notably, Notch pathway elements were consistently downregulated or upregulated in ASPH negative vs. positive breast cancer patients (Additional file 1: Figure S1F-G). ASPH’s expression level positively correlated with active Notch1, ADAM17 and MMPs (*r* ≥ 0.6, *p* < 0.001, 2-sided) (Additional file 1: Figure S1H-I, K). Accordingly, active Notch1 expression level positively correlated with regulator ADAM17 and downstream MMPs (Additional file 1: Figure Fig.S1J, L).

To decipher protein-protein interaction established by ASPH, co-immunoprecipitation (co-IP) and Western blots were conducted. The structure of ASPH protein consists of cytoplasmic, transmembrane, luminal region and catalytic domain (containing the catalytic site M^670^HPGTH^675^) (Additional file 2: Figure S2A). ASPH physically interacts with Notch receptors, ligands (JAGs) (Fig. 1b) or regulators ADAM (A Disintegrin And Metallopeptidase Domain) 10/17 (Fig. 1c). HydroX domain but not Tetratricopeptide repeats (TPRs) of ASPH is required for interaction with Notch ECD (extracellular domain) (Additional file 2: Figure S2B). ASPH enhanced physical interaction between Notch and JAG (Fig. 1b) to stabilize Notch receptors, ligands and regulators, strengthen ligand-receptor binding, and thus boost S2 cleavage of Notch receptors executed by ADAM10/17 α-secretase complex. Consequently, in both ligand-dependent and ligand-independent (ADAM10/17 dependent) manners, Notch signaling is activated by ASPH as clarified by Western blot (Fig. 1d, Additional file 2: Figure S2C-F; Additional file 3: Figure S3A) and luciferase reporter (Fig. 1e, Additional file 2: Figure S2G-I) assays in MDA-MB-231, T47D, MDA-MB-468, and BT474 cells.

As ASPH’s function depends on β-hydroxylase activity, the 3rd generation SMIs against enzymatic activity of ASPH were characterized (Additional file 2: Figure S2J) [2, 11]. MO-I-1182 exhibited a dose-dependent effect on cell viability (Additional file 2: Figure S2K). Can ASPH mediated pro-oncogenic properties be reversed/blocked by SMIs of β-hydroxylase activity? Indeed, MO-I-1182 inhibited ASPH mediated Notch signaling activation, as indicated by luciferase reporter (Fig. 1E**;** Additional file 2: Figure S2L) and confirmed by Western Blot showing downregulation of activated receptors (Notch1), ligands (JAGs), regulators (ADAM9/10/17) and downstream target genes (Fig. 1d**;** Additional file 2: Figure S2M; Additional file 3: Figure S3A) in MDA-MB-231, T47D, MDA-MB-468, and BT474 cells.

To elicit how ASPH-Notch axis mediates aggressive cellular behaviors, γ-secretase complex inhibitor DAPT was employed. In MDA-MB-231 and MDA-MB-468 stably expressing WT-ASPH but not empty vector, MO-I-1182 abrogated while DAPT reduced ECM degradation/remodeling (Fig. 1f; Additional file 3: Figure S3D), 3-D invasion (Fig. 1g; Additional file 3: Figure S3B), EMT (Additional file 3: Figure S3G) and stemness (Fig. 1h; Additional file 3: Figure S3I-L). Endogenous ASPH induced ECM degradation/remodeling (Additional file 3: Figure S3C, E), EMT (Additional file 3: Figure S3F) and stemness (Additional file 3: Figure S3H, M-N) were impaired by both MO-I-1182 and DAPT in T47D and BT474. ASPH KO cells showed no response to MO-I-1182 or DAPT.

To clarify ASPH’s pro-oncogenic properties, in vitro metastasis assay was newly developed (Fig. 1i). This system mimics how breast cancer cells invade through local basement membrane at primary site, intravasate into/extravasate out of vasculature system (transendothelial migration through HUVECs), subsequently penetrate through basement membrane (3-D invasion) and consequently form metastatic colonization/outgrowth (mammosphere formation) at distant sites. Accordingly, both MO-I-1182 and DAPT mitigated in vitro metastasis (Fig. 1j-k).

### ASPH guides breast cancer cells to secrete exosomes delivering pro-oncogenic/pro-invasive cargoes

Notch signaling pathway promotes exosomal release [26]. We proposed, through activating Notch cascade, ASPH could instruct breast cancer cells to synthesize/release designated exosomes, leading to dissemination and metastatic outgrowth. To clarify how ASPH mediated featured exosomes could spur tumor progression, exosomes were extracted and purified from breast cancer cell lines by ultracentrifugation (Additional file 4: Figure S4A). Released exosomes (with a density at 1.10–1.12 g/mL on sucrose gradients) were characterized by morphology (size and shape) under transmission electron microscopy, as well as expression of classic (e.g., CD9, CD63, CD81) and specific (e.g., MMP2) biomarkers (Additional file 4: Figure S4B-F).

**Fig. 2:**
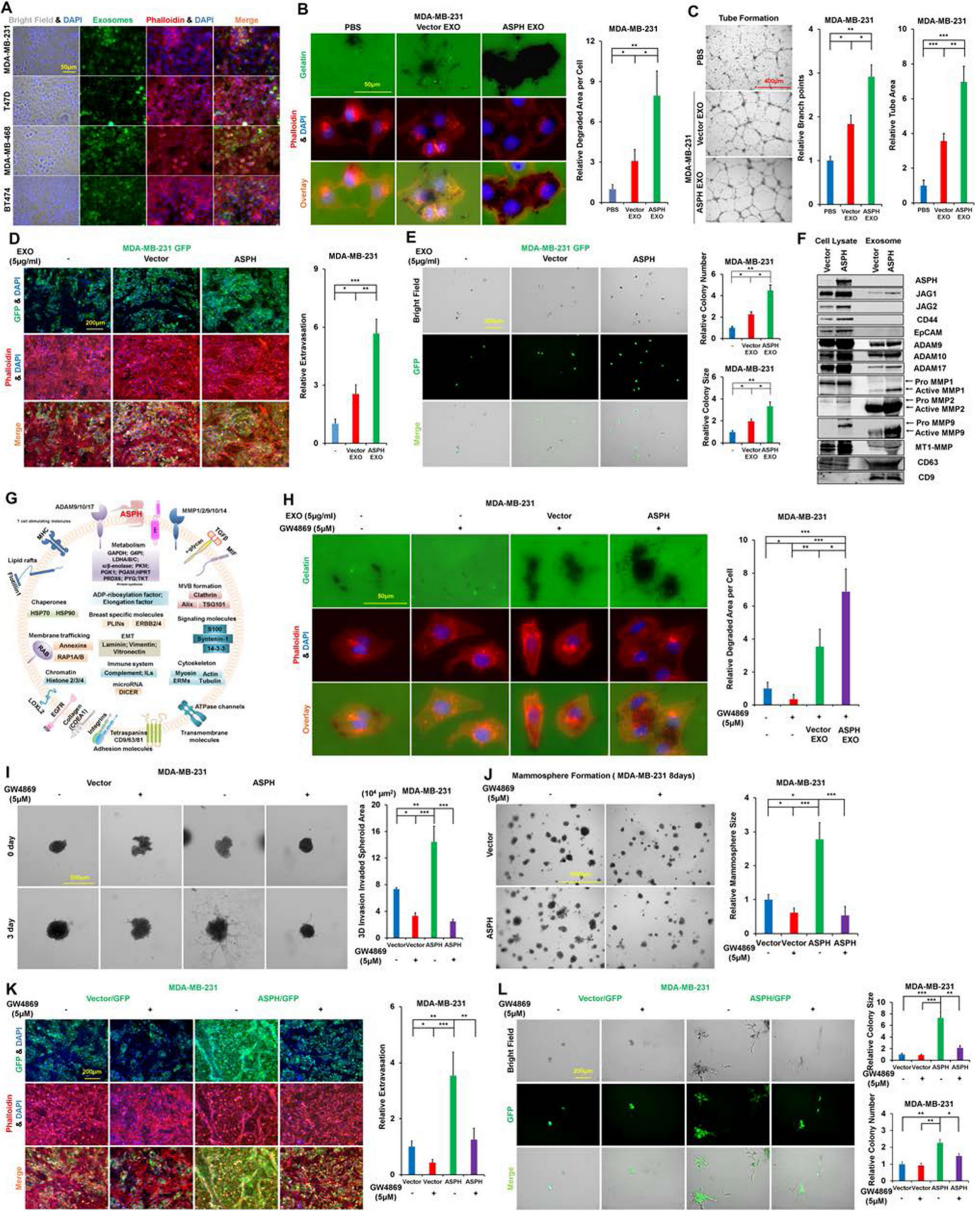
ASPH guides breast cancer cells to secrete pro-oncogenic/pro-metastatic extracellular vesicles. (**a**) Exosomes secreted by MDA-MB-231 cells stably overexpressing ASPH were actively taken up by parental breast cancer cells. (**b**) ECM degradation/remodeling in parental cells incubated with exosomes released from MDA-MB-231 expressing vector or ASPH. (**c**) Tube formation of parental cells incubated with exosomes secreted by MDA-MB-231 cells vs. with PBS. (**d**) Transendothelial migration and extravasation; (**e**) Invasion through basement membrane and subsequent mammosphere formation of parental MDA-MB-231 cells incubated with exosomes secreted by MDA-MB-231 expressing vector or ASPH. (**f**) Compared to empty vector, the exosomes (expressing classic biomarkers CD63 and CD9) released by MDA-MB-231 stably expressing WT-ASPH exhibited enrichment of pro-metastatic components activated Notch1, JAG1/2, CD44, EpCAM, MMPs, and ADAMs. (**g**) Representative protein cargoes of extracellular vesicles released by MDA-MB-231 cells expressing WT-ASPH was deciphered with proteomics using Mass Spectrometry. (**h**) GW4869 blocked ECM degradation of parental cells, which was rescued by addition of exosomes secreted by MDA-MB-231 stably expressing ASPH (vector to a much less extent). (**i**) 3-D invasion in response to GW4869. (**j**) Mammosphere formation in response to GW4869. (**k**) Transendothelial migration and extravasation; (**l**) Invasion through basement membrane and subsequent mammosphere formation in response to GW4869. ^*^p < 0.05; ^**^p < 0.01; ^***^p < 0.001

To explore if donors transferred exosomes with commitment could reprogram recipients’ cellular behaviors, breast cancer cells were co-incubated with specific exosomes. Exosomes secreted by more malignant donors (MDA-MB-231 expressing WT-ASPH) were avidly taken up by less malignant recipients (parental MDA-MB-231, MDA-MB-468, T47D and BT474) (Fig. 2a). Exosomes were recruited to ECM degradation sites under immunofluorescent microscopy as confirmed by co-localization of exosome marker CD63 with MMP2 (Additional file 4: Figure S4G-J). Sorted exosomes delivered from donors strengthened ECM degradation/remodeling (Fig. 2B**;** Additional file 4: Figure S4K-M) and mammosphere formation of recipients.

To elucidate if exosomes could participate in ASPH mediated angiogenesis and/or lymphogenesis, tube formation (in vitro angiogenesis) was performed. Exosomes secreted by MDA-MB-231 and MDA-MB-468 stably expressing WT-ASPH (vector to a much less extent) enhanced tube formation (Fig. 2c; Additional file 4: Figure S4N). Furthermore, WT-ASPH (vector to a much less extent) guided MDA-MB-231 to release exosomes to boost in vitro metastasis (Fig. 2d-e).

To decipher protein cargoes harbored by exosomes, Western blot and proteomics were performed. Compared to empty vector, ASPH guided MDA-MB-231 to secrete exosomes carrying pro-invasive/pro-metastatic components including active Notch receptor, ligand, regulators ADAMs and downstream MMPs (Fig. 2f; Additional file 4: Figure S4O-P). These elements were upregulated in the less malignant recipients (T47D and BT474) upon taking-up the specific exosomes from the more malignant donor (MDA-MB-231 expressing WT-ASPH). As identified by proteomics, compared to vector control, exogenous WT-ASPH enabled breast cancer cells to release sorted exosomes that transfer various proteins involved in multiple processes, such as metabolism, invasion, metastasis and immunosuppression (Fig. 2g; Additional file 4: Figure S4Q).

Neutral sphingomyelinase 2 (N-SMase 2) mediates cellular stress-induced biologically active sphingolipids (e.g., ceramide) and is vital for exosomes synthesis/release [27]. We proposed N-SMase 2 is required for exosomal polarization to promote breast cancer pathogenesis [28]. To clarify the role of N-SMase 2 in ASPH induced aggressive phenotypes, GW4869 was used to inhibit N-SMase activity. In MDA-MB-231 and MDA-MB-468, exogenous ASPH mediated ECM degradation/remodeling (Additional file 5: Figure S5A, C), 3-D invasion (Fig. 2; Additional file 5: Figure S5E), stemness (Fig. 2j**;** Additional file 5: Figure S5F) and in vitro metastasis (Fig. 2k-l) were undermined by GW4869. Endogenous ASPH induced ECM degradation/remodeling (Additional file 5: Figure S5B, D) and stemness (Additional file 5: Figure S5G) were attenuated by N-SMase inhibitor in T47D and BT474. ASPH KO cells showed no response.

Importantly, reduction in ECM degradation/remodeling of parental MDA-MB-231 and MDA-MB-468 by N-SMase inhibitor was rescued by re-addition of exosomes secreted by MDA-MB-231 cells expressing WT-ASPH (empty vector to a much less extent) (Fig. 2h).

### Exosomes act as an outlet of notch-upregulated MMPs production; MMPs function as an executor responsible for ASPH mediated aggressive malignant phenotypes in breast cancer

ASPH activates Notch signaling to upregulate downstream target genes, in particular MMPs, which have an outlet, i.e., to be packaged into exosomes. Simultaneously, ASPH stabilizes and upregulates ADAMs. We hypothesized MMPs (ADAMs) as executive effectors of exosomes are pivotal for ASPH mediated aggressive phenotypes of breast cancer cells.

**Fig. 3:**
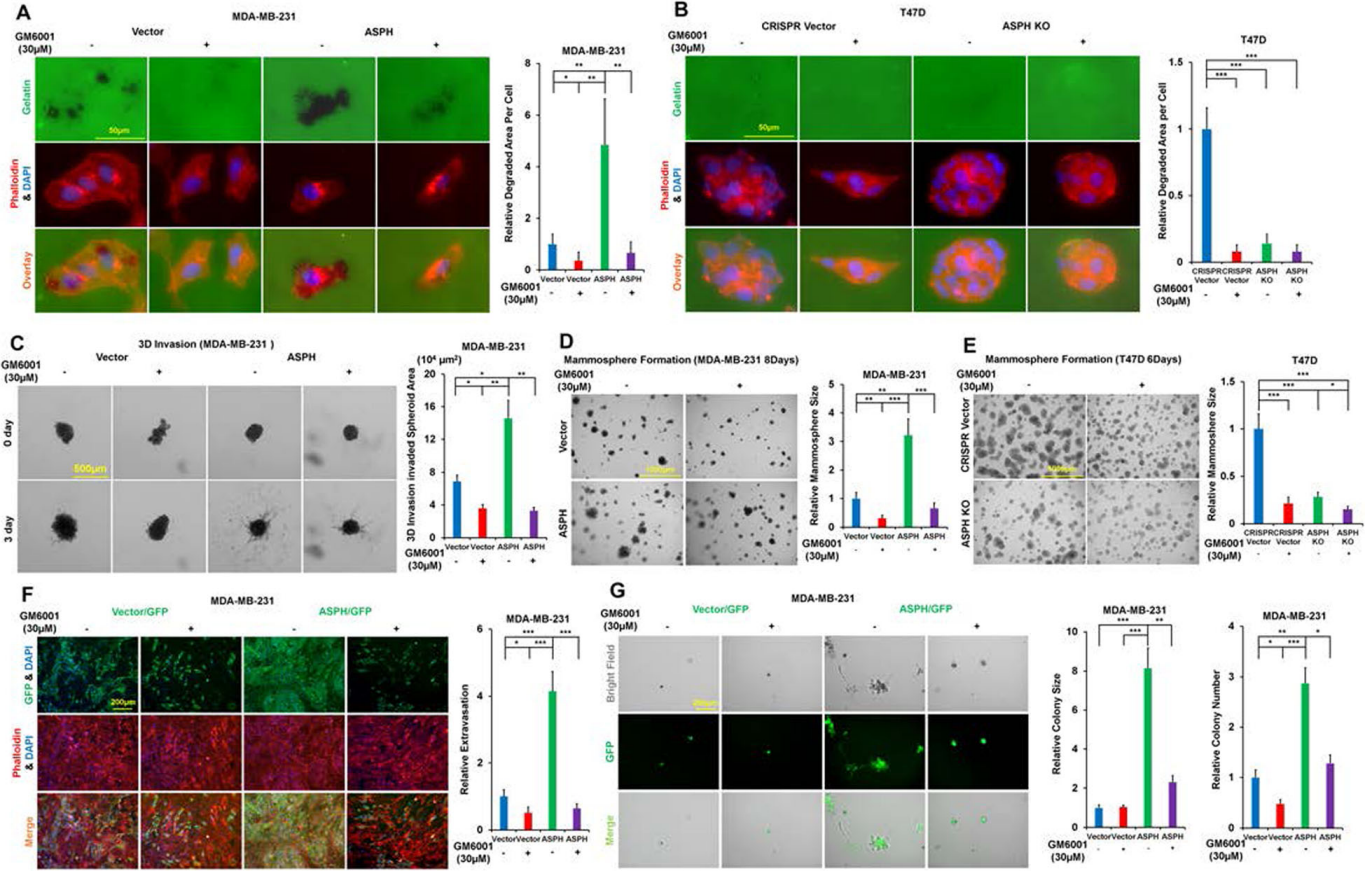
Exosomes act as an outlet of MMPs; MMPs function as a direct executor for exosomes, contributing to ASPH mediated aggressive malignant phenotypes in breast cancer. (**a**-**b**) Invadopodia formation and ECM degradation/remodeling in response to inhibition of MMPs activities using GM6001. (**c**) 3-D invasion in response to GM6001. (**d-e**) Mammosphere formation in response to GM6001. (**f**) Transendothelial migration (intravasation/extravasation); (**g**) Invasion through basement membrane and subsequent mammosphere formation in response to GM6001. ^*^p < 0.05; ^**^p < 0.01; ^***^p < 0.001

To confirm MMPs contribute to ASPH-induced pathogenesis, GM6001, a broad-spectrum inhibitor of metalloproteinases was employed. ASPH enhanced ECM degradation/remodeling (Fig. 3a; Additional file 6: Figure S6A), 3-D invasion (Fig. 3c; Additional file 6: Figure S6C), stemness (Fig. 3d; Additional file 6: Figure S6D) and in vitro metastasis (Fig. 3F-G) were impeded by GM6001 in MDA-MB-231 and MDA-MB-468. Endogenous ASPH induced ECM degradation/remodeling (Fig. 3b**;** Additional file 6: Figure S6B) and stemness (Fig. 3e; Additional file 6: Figure S6E) were diminished by GM6001 in T47D and BT474. ASPH KO cells showed no response.

### ASPH promotes breast cancer metastasis in vivo

**Fig. 4:**
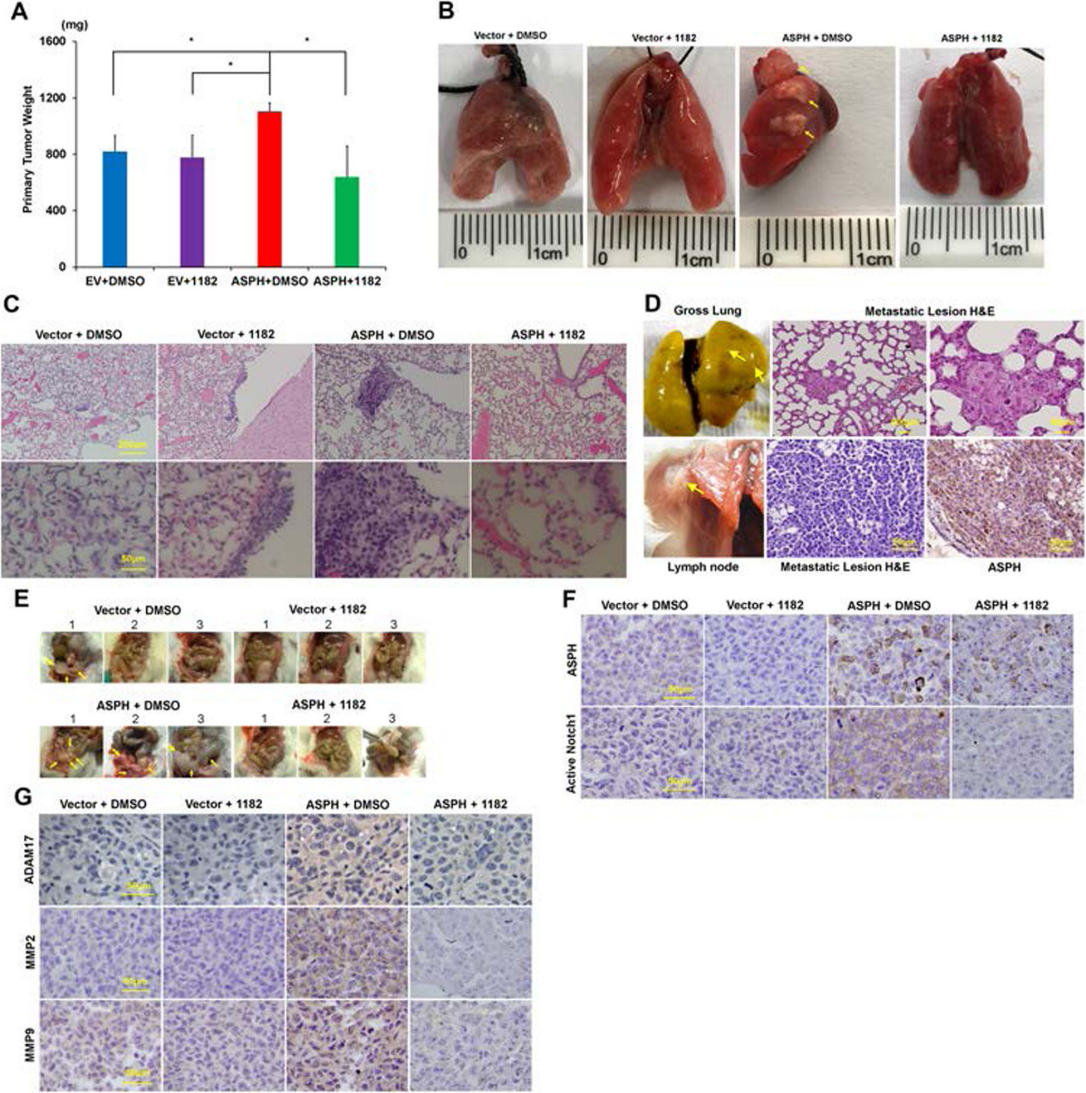
ASPH activates Notch signaling pathway to spur metastasis in orthotopic murine model. (**a**) Compared to empty vector, MDA-MB-231 cells stably expressing ASPH exhibited high tumorigenicity in orthotopic model (*n* = 5/group). Increased primary tumor weight illustrated tumor development was bolstered by ASPH. Anti-tumor effect of MO-I-1182 was notable in tumors generated from MDA-MB-231 cells with exogenous ASPH. ^*^p < 0.05; ^**^p < 0.01. (**b**) Gross appearance of the lungs derived from representative mice in orthotopic model. Metastatic lesions were highlighted with yellow arrows. (**c**) Histologic characteristics of pulmonary metastases. (**d**) Gross appearance and histologic characteristics of (Upper) pulmonary and (Lower) axillary lymph nodes metastatic lesions of two representative mice in orthotopic model. Metastatic lesions maintained high expression of ASPH. These mice were orthotopically injected with MDA-MB-231 stably expressing ASPH and treated with DMSO. (**e**) Gross appearance of the invaded organs by primary breast cancer derived from representative mice in orthotopic model (n = 5/group). Metastatic lesions were highlighted with yellow arrows. (F-G) Expression profiling of key components in Notch signal pathway, including activated Notch1 (ICD), ADAM17, downstream MMPs, was downregulated by the SMI. ^*^p < 0.05; ^**^p < 0.01

To illustrate if ASPH-Notch-ADAMs/MMPs pathway promotes metastasis of breast cancer in vivo, MDA-MB-231 cells stably expressing empty vector vs. ASPH were injected into the 4th mammary fat pad or tail vein of immunodeficient mice. Mice were sacrificed 4 weeks later, and tissues analyzed for micro−/macro-metastases. Compared to Vector, exogenous ASPH generated more aggressive tumors as elicited by both increased weight of primary tumor (Fig. 4a) and widespread metastases to the lungs, lymph nodes, spleen, intestine (colon), mesentery, or liver (Fig. 4b-e) in orthotopic murine models.

As the SMI against β-hydroxylase activity reverses pro-oncogenic properties of ASPH in vitro, we assessed its anti-metastatic effects in vivo*.* Indeed, the size/weight of primary tumors as well as the number/size of metastatic lesions were significantly blocked by MO-I-1182 (10 mg/kg, i.p., every other day) in NSG mice of orthotopic models (Fig. 4a-c, e). Exogenous ASPH activated Notch signaling pathway in vivo was substantially blunted by the SMI as confirmed by downregulation of active Notch receptor/ligand/regulator, and downstream MMPs (Fig. 4f-g).

**Fig. 5:**
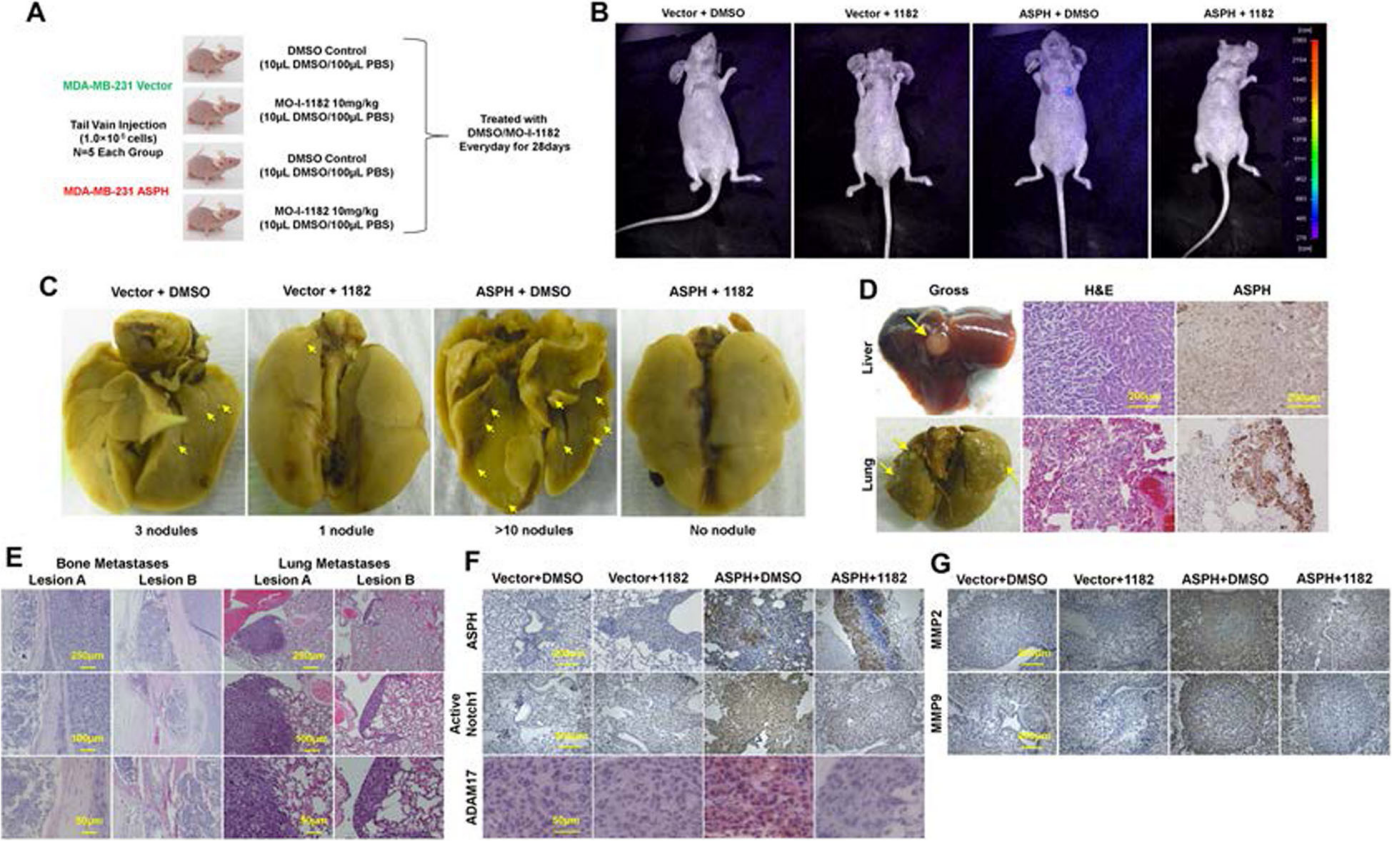
Compared to empty vector, WT-ASPH significantly enhanced metastatic capability of MDA-MB-231 cells, which was efficiently reversed by the SMI in experimental pulmonary metastatic (tail vein injection) murine model. (**a**) Experimental design and Therapeutic protocol for tail vein injection model (n = 5/group). (**b**) Using fluorescent imaging system to detect potential pulmonary metastasis in mice from different groups of tail vein injection model. (**c**) Gross appearance and histologic characteristics of the lungs derived from representative mice in tail vein injection model. Metastatic lesions were highlighted with yellow arrows. (**d**) Gross appearance and histopathologic characteristics of (Upper) hepatic and (Bottom) pulmonary metastatic lesions of a representative mouse in ASPH+DMSO group of tail vein injection model. Noted the metastatic lesions also maintain high expression of ASPH. This animal was euthanized at the 7th weeks. (**e**) Histologic characteristics of bone and lung lesions in a representative mouse. The mouse was tail vein injected with ASPH overexpressing MDA-MB-231 cells and treated with DMSO. (**f**-**g**) Expression profiling of key components in Notch signaling pathways, including activated Notch1, ADAM17 and downstream MMPs, was substantially downregulated by SMI. ^*^p < 0.05; ^**^p < 0.01

In tail vein injection models, BALB/c athymic nude mice were treated with the SMI vs. DMSO (Fig. 5a). Metastatic lesions in the lungs, liver and bone (Fig. 5b-e) formed by MDA-MB-231 stably expressing ASPH and treated with DMSO were substantially intensified compared to Vector. Breast cancer cells highly expressing ASPH accelerated tumor development and progression. Aggressive malignant phenotypes, including the number of micro−/macro-metastatic pulmonary lesions and vasculature invasion, were substantially blocked by MO-I-1182 (10 mg/kg, i.p., every other day) (Fig. 5b-c). Exogenous ASPH activated Notch pathway in vivo was reversed by the SMI as confirmed by downregulation of active Notch receptor/ligand/regulator, and downstream MMPs (Fig. 5f-g).

### ASPH is upregulated with disease progression and predicts prognosis

To decipher differential expression of ASPH in tumors compared with adjacent non-malignant breast tissue, IHC was conducted in 141 de-identified breast cancer patients. Their demographic and clinical features are described in Additional file 7: Table S1. ASPH was detected in 90.1% of breast cancer patients, with a negative, low, moderate or high expression rate of 9.9, 6.4, 43.3, and 41.1%, respectively. The mean staining intensity score was 2.0 (0: negative, 1: weak, 2: intermediate, 3: strong). ASPH was undetectable in normal breast, mastitis or benign breast tumor (intraductal papilloma or fibroadenoma). ASPH was detectable at early stage breast neoplasm, i.e., preinvasive DISC (ductal carcinoma in situ), substantially enhanced from IDC (invasive ductal carcinoma) to advanced/spontaneously metastatic breast cancer.

**Fig. 6:**
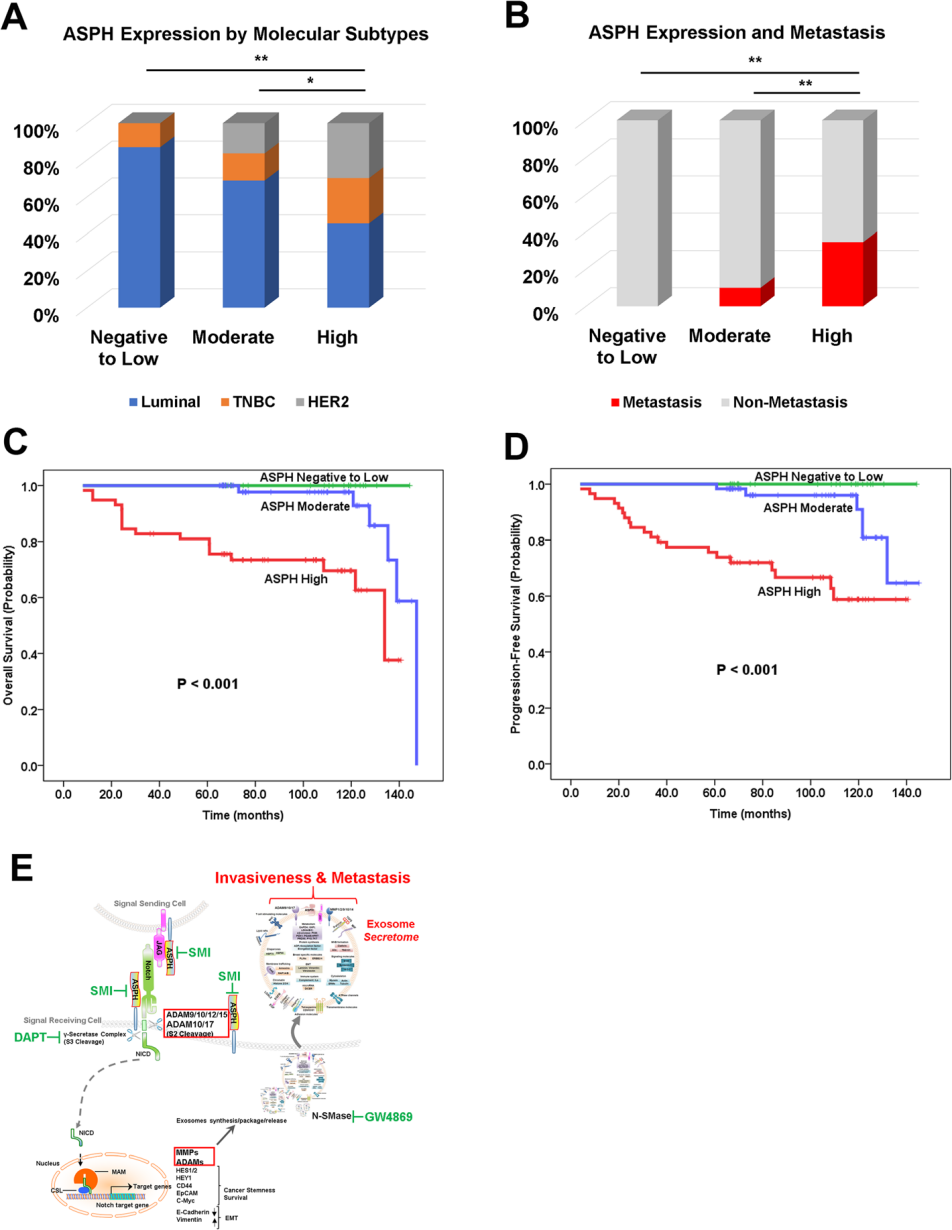
ASPH expression predicts prognosis of breast cancer patients. (**a**) ASPH expression in different molecular subtypes. (**b**) ASPH expression related to incidence of recurrence/progression or metastasis. (**c**-**d**) ASPH expression levels correlated with OS or PFS of breast cancer patients. (**e**) Hypothesized role of ASPH-Notch axis in pathogenesis of breast cancer. ^*^p < 0.05; ^**^p < 0.01

To demonstrate if ASPH expression levels could predict prognosis of breast cancer patients, Kaplan–Meier plots and Cox proportional hazards regression models were applied. Compared to negative-low levels, moderate-high levels of ASPH conferred more devastating molecular subtypes, i.e., triple negative/basal like breast cancer (TNBC) (88.5% vs. 11.5%, respectively) and HER2 amplification (100% vs. 0%, respectively), in contrast to luminal A/B (77.3% vs. 22.7%, respectively) (Fig. 6a). Moderate-high levels of ASPH predicted higher risks of recurrence, progression and metastasis (Fig. 6b), whereas diminished OS and PFS (log-rank test, *Ps* < 0.001) (Fig. 6c-d). Therefore, ASPH is proposed to be a potential diagnostic and prognostic marker for breast cancer.

## Discussion

ASPH directly interacts with Notch/JAGs and ADAM10/17, subsequently activates Notch signaling pathway, instructs synthesis/secretion of pro-oncogenic exosomes in an attempt to prepare pre-metastatic niche, through enhancing EMT, 3-D invasion, angiogenesis (tube formation), stemness (mammosphere formation and cancer stem cell markers upregulation), transendothelial migration (intravasation/extravasation) and eventual metastatic colonization/outgrowth at distant sites,

We examined ASPH expression profiling in several human breast cancer cell lines. However, due to a limited number of cell lines being screened, a selection bias cannot be excluded. Stable MDA-MB-231 cell lines (with a certain level of endogenous ASPH expression) were established to knock-out ASPH using CRISPR-CAS9 system. Compared to vector, ASPH KO significantly reduced aggressive phenotypes (2D/3D invasion, mammosphere formation, angiogenesis and in vitro metastasis) of MDA-MB-231 cells.

In the presence of β-hydroxylase activity, in a ligand-dependent manner, ASPH binding kto both Notch ligands (JAG/DLL) on signal sending cells and Notch receptors on signal receiving cells enhances stability of and interaction between Notch receptors and ligands, subjected to two consecutively proteolytic cleavages of Notch receptors [8, 10]. The metalloproteases ADAM10/17 catalyze S2 cleavage and generate a substrate for S3 cleavage, which is conducted by γ-secretase complex. In a ligand-independent manner, ASPH physically interacts with and increases stability of ADAM10/17 to facilitate the S2 cleavage of Notch receptors. The S2/S3 cleavages lead to the release of Notch intracellular domain (NICD), which shuttles to nucleus and interacts with DNA-binding proteins CSL [CBF1, Su(H) and LAG-1]. Co-activator Mastermind (Mam) and other transcription factors are recruited to CSL complex; and simultaneously, co-repressors (Co-Rs) are dismissed. Thus, the Notch signaling is ON and downstream target genes are upregulated. MMPs, ADAMs, HES1/2, HEY1, c-Myc, CD44, EpCAM and Vimentin (downregulation of E-cadherin) engage in survival, stemness, EMT, invasion, ECM degradation/remodeling, angiogenesis, intravasation/extravasation, and metastatic colonization in breast cancer. SMIs block the catalytic site of ASPH, resulting in decreased β-hydroxylase activity by 80–95% and reduced activation of Notch pathway. Consequently, ASPH-Notch axis is OFF and exosomes mediated multifaceted metastasis is abolished (Fig. 6e). Consistently, ASPH-induced malignant phenotypes are lessened by γ-secretase inhibitor DAPT.

Pathological exosomes render aggressively malignant phenotypes. However, regulatory control of exosomal synthesis, release and function remains mysterious. Based on proteomics, ASPH is proposed to guide sorting/packaging of exosomes via upregulating RAB proteins, e.g., RAB10 [29], RAB11 [30] and RAB27, which localize to exocytic/endocytic compartments and regulate intracellular vesicle trafficking. ASPH instructs the more malignant breast cancer cells (donors) to synthesize/secrete exosomes delivering designated elements, aiming to reprogram fundamental processes of the less malignant cells (recipients), such as cytoskeleton dynamics, metabolism, stemness and multifaceted metastasis.

ASPH is proposed to guide breast cancer cells to synthesize, release/secrete and deliver metalloproteinases enriched exosomes. Inhibiting ASPH-guided pro-oncogenic exosomes secretion with N-SMase inhibitor GW4869 impaired ECM degradation/remodeling. This inhibition was rescued by re-addition of purified exosomes released from breast cancer cells with exogenous ASPH. Therefore, exosomal cargoes (especially proteinases) confer invasiveness. Exosomes enhance ECM degradation/remodeling, stemness, 3-D invasion and in vitro metastasis. MMPs upregulated by Notch signal and ADAMs stabilized by ASPH are core materials for pro-oncogenic/pro-metastatic exosomes secretome. MMPs/ADAMs act as outlets of ASPH-Notch axis-activation and immediate effectors for ECM degradation/remodeling to initiate multistep metastasis of breast cancer cells. Thus, inhibition of pan-MMPs’ activity with GM6001 also attenuates ASPH mediated aggressive behaviors.

ASPH is detectable in 90.1% of breast cancer patients, but not in inflammatory disease or benign tumor. ASPH is silenced in adult normal breast, but re-emerges at early stage preinvasive DISC and highly expressed in advanced/metastatic breast cancer patients. Compared to negative-low levels, moderate-high levels of ASPH conferred more devastating molecular subtypes (TNBC and HER2 amplified), early relapse, higher risk of distant multiple-organ metastasis, and reduced survival. Importantly, ASPH secreted by cancer cells and delivered by exosomes can be detected in biofluids (e.g., peripheral blood). Recently, a highly sensitive serum-based cancer diagnostics using ELISA has been developed, establishing ASPH (HAAH) as a potential biomarker for early-stage cancer [31]. This pan-cancer biomarker has been identified in multiple malignancies including breast cancer using serum exosomes derived from patients. The overall accuracy of this exosome-enabled ELISA is 93.8%. A large-scale international collaboration will be performed to evaluate potential value of ASPH as a biomarker for cancer diagnosis and prognosis.

Exosomes enable prooncogenic secretome delivering/trafficking for long-distance cell-cell communication. ASPH-Notch axis guides designated exosomes to promote metastatic colonization/outgrowth at distant sites in MDA-MB-231 generated orthotopic and tail vein injection murine models. Primary tumor growth and distant metastases driven and accelerated by ASPH network are specifically/efficiently blocked by the 3rd generation SMI. ASPH-Notch axis results in MMPs/ADAMs mediated ECM degradation/remodeling as a direct executioner for invasiveness. These aggressive phenotypes are reversed by SMIs specifically against ASPH’s β-hydroxylase activity (both in vitro and in vivo). Collectively, to the best of our knowledge, for the first time, this study has revealed a fundamental role of ASPH in propagating invasion, aggressiveness and multi-organ dissemination, thus establishing ASPH as a potential therapeutic target for multifaceted metastasis of breast cancer.

## Supplementary information

**Additional file 1: Figure S1.** ASPH-Notch network components are consistently upregulated or downregulated in tumors derived from breast cancer. (A-B) Expression profiling of ASPH and Notch receptors/ligands in different human breast cancer cell lines. (C-D) Left Panel: Lentiviral transfection and stable overexpression of WT-ASPH or H675Q mutant in MDA-MB-231 and MDA-MB-468 cells. Right Panel: Lentiviral transfection and CRISPR-CAS9 system induced stable knockout (KO) of ASPH in BT474 and T47D cells. (E) Upper: Histopathological characteristics of 6 representative tumors derived from breast cancer patients. Lower: ASPH expression by IHC. (F-G) Consistent downregulation vs. upregulation of Activated Notch1, ADAM17/TACE MMP-2 and MMP-9 in ASPH negative vs. positive tumors in tumor cells compared to adjacent non-malignant tissues (*P* < 0.001, 2-sided paired t test). (H-L) ASPH expression level positively correlated with Activated Notch1, ADAM17, and MMP2; Activated Notch1 expression level positively correlated with ADAM17/TACE and MMP-2 levels in breast cancer patients (*N* = 87). ^*^p < 0.05; ^**^p < 0.01; ^***^p < 0.001.

**Additional file 2: Figure S2.** ASPH activates Notch signaling pathway in breast cancer cells. (A) Domains and functional units of ASPH protein. (B) Aspartyl/Asparaginyl β-hydroxylase (HydroX) domain (containing amino acid 591–744) is required for its interaction with Notch extracellular domain (ECD). Tetratricopeptide repeat (TPR) domain typically contains 34 amino acids. TPR6 contains amino acid 525–548. (C-F) Expression profile of Notch signaling pathway components in MDA-MB-231, T47D, MDA-MB-468 or BT474 cells, respectively. (G-I) Luciferase reporter assays demonstrated the activation of Notch signaling in MDA-MB-231, T47D or BT474 cells in the presence of both full-length (FL) Notch and ASPH. Reporter alone was used as a negative control whereas activated Notch1 (Notch intracellular domain; NICD) alone as a positive control. Notch signaling was active in T47D or BT474 CRISPR vector cells with endogenous ASPH expression but inactivated in CRISPR-CAS9 ASPH KO cells. (J-K) Characterization of the 3rd generation SMIs of ASPH. (J) Candidate parent compounds selected and evaluated as potential inhibitors of ASPH β-hydroxylase activity. Using computer assisted drug design, those compounds were synthesized based on crystal structure of the catalytic site in the C-terminal region of ASPH. (K) Effects of candidate compounds on cell viability. MO-I-1182 had demonstrated a dose-dependent effect over a range of 1.25–10 μM; MO-I-1188, 1149 and 1185 showed little, if any, inhibitory effect. (L) Luciferase reporter assay demonstrated the activation of Notch signaling in MDA-MB-468 in the presence of both FL-Notch and ASPH, which could be efficiently inhibited by both SMI and DAPT. ^*^p < 0.05; ^**^p < 0.01; ^***^p < 0.001.

**Additional file 3: Figure S3.** ASPH promotes 3-D invasion, ECM degradation/remodeling, EMT and stemness in breast cancer cells. (A) Notch signaling components in MDA-MB-468 or BT474 cells in response to SMI. (B) 3-D invasion of MDA-MB-468 cells in response to SMI or DAPT, respectively. (C-E) ECM degradation/remodeling in T47D, MDA-MB-468 or BT474 cells in response to SMI or DAPT, respectively. (F) Expression of epithelial marker E-cadherin in T47D cells in response to SMI and DAPT, respectively. (G) Expression of mesenchymal marker Vimentin in MDA-MB-231 cells in response to SMI or DAPT, respectively. (H-I) Expression of cancer stem cell marker EpCAM in T47D or MDA-MB-468 cells in response to SMI or DAPT, respectively. (J-K) Expression of cancer stem cell marker CD44 in MDA-MB-231 or MDA-MB-468 cells in response to SMI and DAPT, respectively. (L-N) Mammosphere formation of MDA-MB-468, T47D or BT474 cells in response to SMI or DAPT, respectively. ^*^p < 0.05; ^**^p < 0.01; ^***^p < 0.001.

**Additional file 4: Figure S4.** Exosomes secreted by more malignant donor cells dramatically enhanced aggressive phenotypes of less malignant recipient cells. (A) Protocol for exosomes extraction and purification by ultracentrifugation. (B) Transmission electron microscopy of exosomes secreted by MDA-MB-231 cells expressing empty vector and WT-ASPH, respectively. (C-F) Exo-Green labeled exosomes secreted by MDA-MB-231 cells bound to CD9, CD63, CD81 and MMP-2 beads, respectively. PBS served as control. (G) Co-localization of exosomal marker CD63 and ECM degradation machinery executor MMP2 in MDA-MB-231. (H) Co-localization of exosome marker CD63 and ECM degradation machinery MMP2 in HEK293 cells transfected with CD63-eGFP and MMP2-mCherry plasmids. (I-J) Parental MDA-MB-231, T47D, MDA-MB-486 and BT474 cells actively took up exosomes secreted by MDA-MB-231 cells expressing WT-ASPH at invadopodia sites (docking station) as demonstrated by co-localization of CD63 and MMP2. (K-M) ECM degradation/remodeling in parental MDA-MB-468, T47D or BT474 cells incubated with exosomes released from MDA-MB-231 cells stably expressing empty vector or WT-ASPH. (N) Tube formation of parental MDA-MB-231 cells incubated with exosomes secreted by MDA-MB-231 cells stably expressing empty vector vs. WT-ASPH. (O) Exosomes released by MDA-MB-468 cells expressing WT-ASPH exhibited enrichment of pro-metastatic components activated Notch1, JAG1/2; MMPs; and ADAMs.(P) Representative protein cargoes of exosomes and ectosomes released by MDA-MB-231 cells expressing empty vector as deciphered with proteomics using Mass Spectrometry. ^*^p < 0.05; ^**^p < 0.01; ^***^p < 0.001.

**Additional file 5: Figure S5.** Inhibition of exosomes synthesis/release efficiently attenuated ASPH-mediated malignant phenotypes of breast cancer cells. (A-D) ECM degradation/remodeling in MDA-MB-231, T47D, MDA-MB-468 or BT474 cells, respectively, in response to GW4869. (E) 3-D invasion of MDA-MB-468 cells in response to GW4869. (F-G) Mammosphere formation of MDA-MB-468 or BT47 cells in response to GW4869. ^*^p < 0.05; ^**^p < 0.01; ^***^p < 0.001.

**Additional file 6: Figure S6.** Inhibition of MMPs activity dramatically reduced ASPH rendered cellular behaviors in breast cancer. (A-B) ECM degradation/remodeling in MDA-MB-468 or BT474 cells in response to GM6001. (C) 3-D invasion of MDA-MB-468 cells in response to GM6001. (D-E) Mammosphere formation of MDA-MB-468 or BT47 cells in response to GM6001. ^*^p < 0.05; ^**^p < 0.01; ^***^p < 0.001.

**Additional file 7: Table S1.** Demographic and clinical characteristics of breast cancer patients (validation set).

## Data Availability

Not applicable.

## Abbreviations

3-D: three-dimensional
ADAM: A disintegrin and metalloprotease domain
ASPH: Aspartate β-hydroxylase
co-IP: co-immunoprecipitation
Co-Rs: co-repressors
CSL: CBF1, Su(H) and LAG-1
ECD: Extracellular domain
ECM: Extracellular matrix
EGF: Epidermal growth factor
EMT: Epithelial–mesenchymal transition
H&E: hematoxylin and eosin
HUVEC: Human umbilical vein/vascular endothelium
HydroX: Aspartyl/Asparaginyl β-hydroxylase domain
I.P.: Intraperitoneal
ICD: Intracellular domain
IHC: Immunohistochemistry
JAG: Jagged
KO: knockout
MMP: Matrix metalloproteinase
MVBs: Multivesicular bodies
NSG: NOD.Cg-Prkdc^scid^Il2rg^tm1Wjl^/SzJ; NOD-scid IL2Rg^null^
N-SMase: Neutral sphingomyelinase
RAB: Member RAS Oncogene Family
SMI: small molecule inhibitor
TPR: Tetratricopeptide repeat

## References

[1] Siegel RL, Miller KD, Jemal A (2018). Cancer statistics, 2018. CA Cancer J Clin.

[2] Dong X (2015). Aspartate beta-hydroxylase expression promotes a malignant pancreatic cellular phenotype. Oncotarget.

[3] Iwagami Y (2016). Aspartate beta-hydroxylase modulates cellular senescence through glycogen synthase kinase 3beta in hepatocellular carcinoma. Hepatology.

[4] Tomimaru Y (2015). Aspartate-beta-hydroxylase induces epitope-specific T cell responses in hepatocellular carcinoma. Vaccine.

[5] Dinchuk, J. E. *et al.* Aspartyl beta -hydroxylase (Asph) and an evolutionarily conserved isoform of Asph missing the catalytic domain share exons with junctin. *The Journal of biological chemistry* **275**, 39543–39554, doi:10.1074/jbc.M006753200 (2000). 10.1074/jbc.M006753200 10956665

[6] Lawton M, Tong M, Gundogan F, Wands JR, de la Monte SM (2010). Aspartyl-(asparaginyl) beta-hydroxylase, hypoxia-inducible factor-alpha and notch cross-talk in regulating neuronal motility. Oxidative Med Cell Longev.

[7] de la Monte SM (2006). Aspartyl-(asparaginyl)-beta-hydroxylase regulates hepatocellular carcinoma invasiveness. J Hepatol.

[8] Cantarini MC (2006). Aspartyl-asparagyl beta hydroxylase over-expression in human hepatoma is linked to activation of insulin-like growth factor and notch signaling mechanisms. Hepatology.

[9] Artavanis-Tsakonas S, Rand MD, Lake RJ (1999). Notch signaling: cell fate control and signal integration in development. Science.

[10] Chung W (2016). Activation of signal transduction pathways during hepatic oncogenesis. Cancer Lett.

[11] Aihara A (2014). A cell-surface beta-hydroxylase is a biomarker and therapeutic target for hepatocellular carcinoma. Hepatology.

[12] Wands JR, Kim M (2014). WNT/beta-catenin signaling and hepatocellular carcinoma. Hepatology.

[13] Tomimaru Y (2013). Upregulation of T-cell factor-4 isoform-responsive target genes in hepatocellular carcinoma. Liver international : official journal of the International Association for the Study of the Liver.

[14] Ince N, de la Monte SM, Wands JR (2000). Overexpression of human aspartyl (asparaginyl) beta-hydroxylase is associated with malignant transformation. Cancer Res.

[15] Lavaissiere L (1996). Overexpression of human aspartyl (asparaginyl)beta-hydroxylase in hepatocellular carcinoma and cholangiocarcinoma. J Clin Invest.

[16] Wang K (2010). Overexpression of aspartyl-(asparaginyl)-beta-hydroxylase in hepatocellular carcinoma is associated with worse surgical outcome. Hepatology.

[17] Maeda T (2003). Antisense oligodeoxynucleotides directed against aspartyl (asparaginyl) beta-hydroxylase suppress migration of cholangiocarcinoma cells. J Hepatol.

[18] Sepe Paul S, Lahousse Stephanie A, Gemelli Brad, Chang Howard, Maeda Takashi, Wands Jack R, de la Monte Suzanne M (2002). Role of the Aspartyl-Asparaginyl-β-Hydroxylase Gene in Neuroblastoma Cell Motility. Laboratory Investigation.

[19] Luu M (2009). Prognostic value of aspartyl (asparaginyl)-beta-hydroxylase/humbug expression in non-small cell lung carcinoma. Hum Pathol.

[20] Wang J (2007). Prognostic value of humbug gene overexpression in stage II colon cancer. Hum Pathol.

[21] Gundogan F (2007). Role of aspartyl-(asparaginyl) beta-hydroxylase in placental implantation: relevance to early pregnancy loss. Hum Pathol.

[22] Muralidharan-Chari V, Clancy JW, Sedgwick A, D’Souza-Schorey C (2010). Microvesicles: mediators of extracellular communication during cancer progression. J Cell Sci.

[23] Peinado H (2012). Melanoma exosomes educate bone marrow progenitor cells toward a pro-metastatic phenotype through MET. Nat Med.

[24] Hoshino D (2013). Exosome secretion is enhanced by invadopodia and drives invasive behavior. Cell Rep.

[25] Mathivanan S, Ji H, Simpson RJ (2010). Exosomes: extracellular organelles important in intercellular communication. J Proteome.

[26] Boelens MC (2014). Exosome transfer from stromal to breast cancer cells regulates therapy resistance pathways. Cell.

[27] Shamseddine AA, Airola MV, Hannun YA (2015). Roles and regulation of neutral sphingomyelinase-2 in cellular and pathological processes. Adv Biol Regul.

[28] Ogretmen B, Hannun YA (2004). Biologically active sphingolipids in cancer pathogenesis and treatment. Nat Rev Cancer.

[29] English AR, Voeltz GK (2013). Rab10 GTPase regulates ER dynamics and morphology. Nat Cell Biol.

[30] Savina A, Fader CM, Damiani MT, Colombo MI (2005). Rab11 promotes docking and fusion of multivesicular bodies in a calcium-dependent manner. Traffic.

[31] Semenuk, M.A., Cifuentes, A.S., Ghanbari, E.R., Lebowitz, M.S., Ghanbari, H.A.. Improved Detection of Cancer Specific Serum Exosomal Aspartyl (Asparaginyl) beta Hydroxylase (HAAH). Cancer Res. 2017; 77 (13 Suppl): Abstract #723.

