# Supplementary figures and images for: ASPH-notch Axis guided Exosomal delivery of Prometastatic Secretome renders breast Cancer multi-organ metastasis
Source: Mol Cancer. 2019 Nov 7;18:156. doi: 10.1186/s12943-019-1077-0 (PMC6836474; doi:10.1186/s12943-019-1077-0)

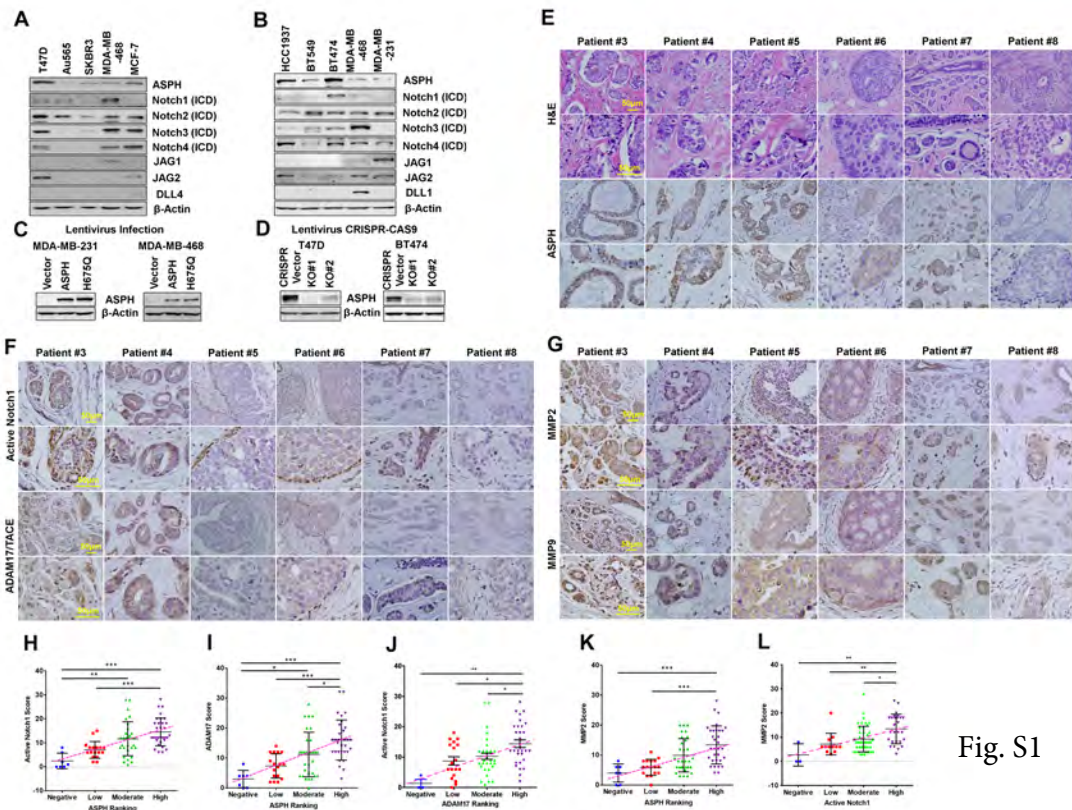

Fig. S1

Supplement: Supplementary file 1 — Additional file 1: Figure S1. ASPH-Notch network components are consistently upregulated or downregulated in tumors derived from breast cancer. (A-B) Expression profiling of ASPH and Notch receptors/ligands in different human breast cancer cell lines. (C-D) Left Panel: Lentiviral transfection and stable overexpression of WT-ASPH or H675Q mutant in MDA-MB-231 and MDA-MB-468 cells. Right Panel: Lentiviral transfection and CRISPR-CAS9 system induced stable knockout (KO) of ASPH in BT474 and T47D cells. (E) Upper: Histopathological characteristics of 6 representative tumors derived from breast cancer patients. Lower: ASPH expression by IHC. (F-G) Consistent downregulation vs. upregulation of Activated Notch1, ADAM17/TACE MMP-2 and MMP-9 in ASPH negative vs. positive tumors in tumor cells compared to adjacent non-malignant tissues (P < 0.001, 2-sided paired t test). (H-L) ASPH expression level positively correlated with Activated Notch1, ADAM17, and MMP2; Activated Notch1 expression level positively correlated with ADAM17/TACE and MMP-2 levels in breast cancer patients (N = 87). *p < 0.05; **p < 0.01; ***p < 0.001. [file 12943_2019_1077_MOESM1_ESM.pdf]

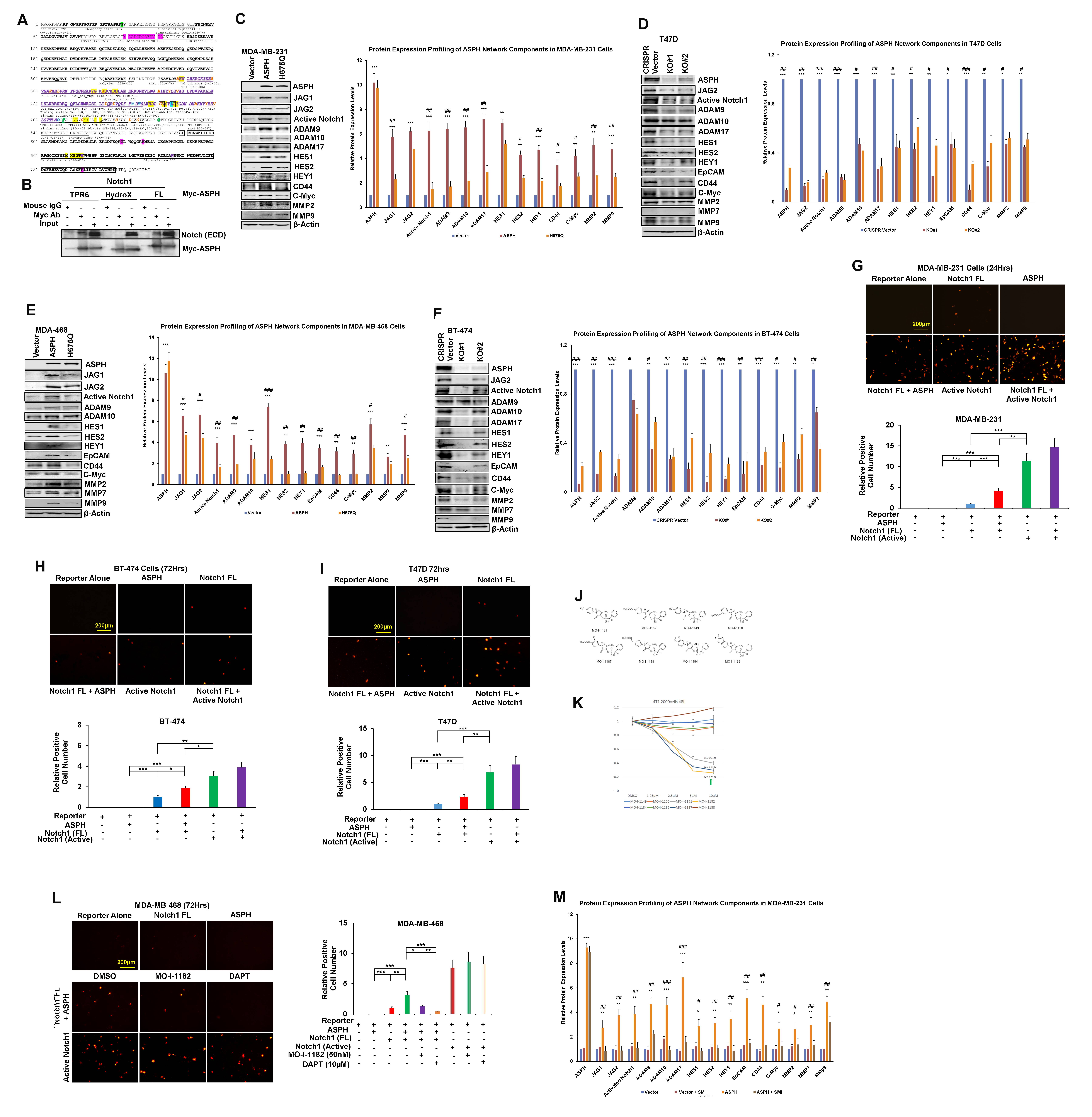

Supplement: Supplementary file 2 — Additional file 2: Figure S2. ASPH activates Notch signaling pathway in breast cancer cells. (A) Domains and functional units of ASPH protein. (B) Aspartyl/Asparaginyl β-hydroxylase (HydroX) domain (containing amino acid 591–744) is required for its interaction with Notch extracellular domain (ECD). Tetratricopeptide repeat (TPR) domain typically contains 34 amino acids. TPR6 contains amino acid 525–548. (C-F) Expression profile of Notch signaling pathway components in MDA-MB-231, T47D, MDA-MB-468 or BT474 cells, respectively. (G-I) Luciferase reporter assays demonstrated the activation of Notch signaling in MDA-MB-231, T47D or BT474 cells in the presence of both full-length (FL) Notch and ASPH. Reporter alone was used as a negative control whereas activated Notch1 (Notch intracellular domain; NICD) alone as a positive control. Notch signaling was active in T47D or BT474 CRISPR vector cells with endogenous ASPH expression but inactivated in CRISPR-CAS9 ASPH KO cells. (J-K) Characterization of the 3rd generation SMIs of ASPH. (J) Candidate parent compounds selected and evaluated as potential inhibitors of ASPH β-hydroxylase activity. Using computer assisted drug design, those compounds were synthesized based on crystal structure of the catalytic site in the C-terminal region of ASPH. (K) Effects of candidate compounds on cell viability. MO-I-1182 had demonstrated a dose-dependent effect over a range of 1.25–10 μM; MO-I-1188, 1149 and 1185 showed little, if any, inhibitory effect. (L) Luciferase reporter assay demonstrated the activation of Notch signaling in MDA-MB-468 in the presence of both FL-Notch and ASPH, which could be efficiently inhibited by both SMI and DAPT. *p < 0.05; **p < 0.01; ***p < 0.001. [file 12943_2019_1077_MOESM2_ESM.jpg]

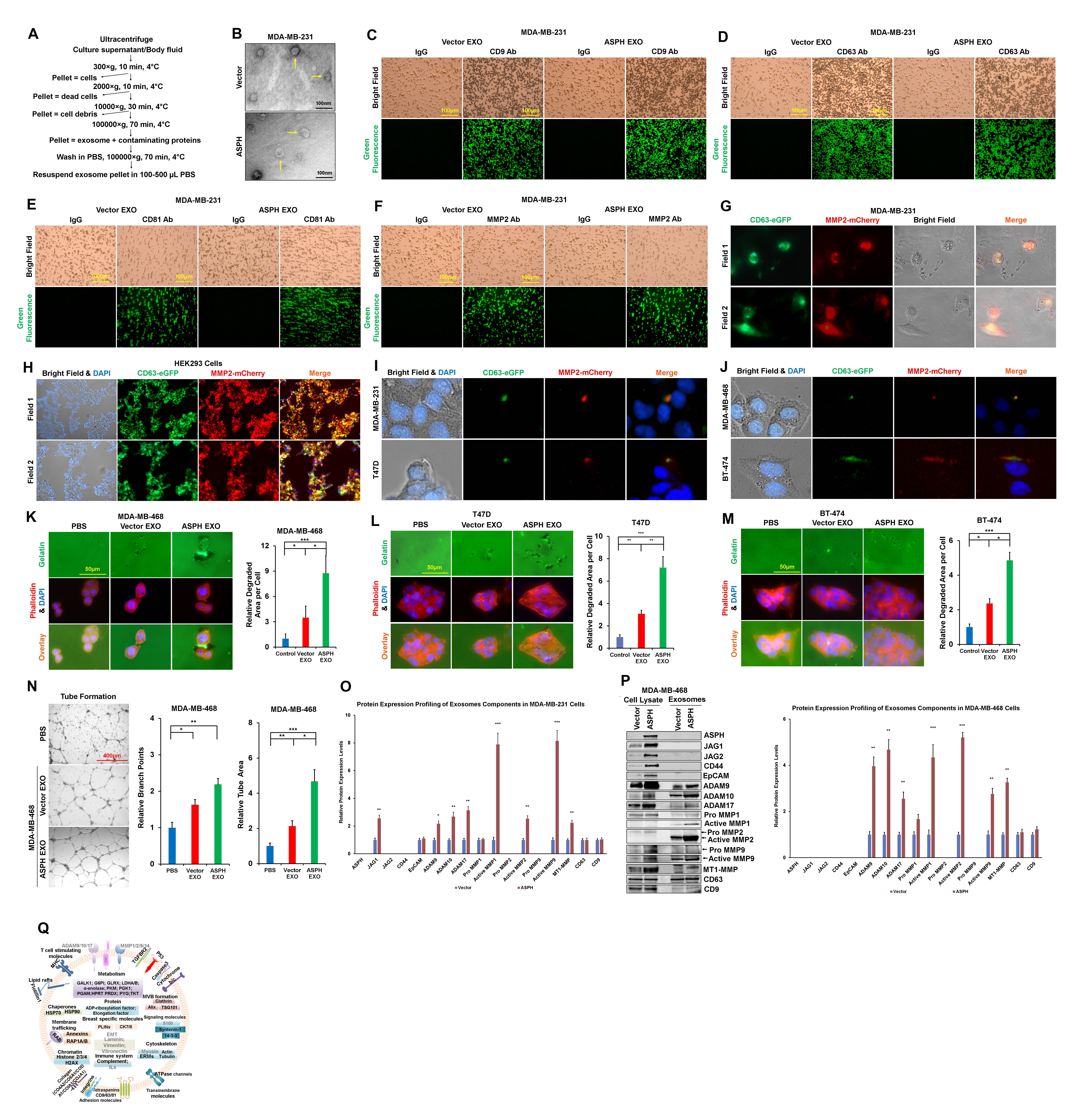

Supplement: Supplementary file 4 — Additional file 4: Figure S4. Exosomes secreted by more malignant donor cells dramatically enhanced aggressive phenotypes of less malignant recipient cells. (A) Protocol for exosomes extraction and purification by ultracentrifugation. (B) Transmission electron microscopy of exosomes secreted by MDA-MB-231 cells expressing empty vector and WT-ASPH, respectively. (C-F) Exo-Green labeled exosomes secreted by MDA-MB-231 cells bound to CD9, CD63, CD81 and MMP-2 beads, respectively. PBS served as control. (G) Co-localization of exosomal marker CD63 and ECM degradation machinery executor MMP2 in MDA-MB-231. (H) Co-localization of exosome marker CD63 and ECM degradation machinery MMP2 in HEK293 cells transfected with CD63-eGFP and MMP2-mCherry plasmids. (I-J) Parental MDA-MB-231, T47D, MDA-MB-486 and BT474 cells actively took up exosomes secreted by MDA-MB-231 cells expressing WT-ASPH at invadopodia sites (docking station) as demonstrated by co-localization of CD63 and MMP2. (K-M) ECM degradation/remodeling in parental MDA-MB-468, T47D or BT474 cells incubated with exosomes released from MDA-MB-231 cells stably expressing empty vector or WT-ASPH. (N) Tube formation of parental MDA-MB-231 cells incubated with exosomes secreted by MDA-MB-231 cells stably expressing empty vector vs. WT-ASPH. (O) Exosomes released by MDA-MB-468 cells expressing WT-ASPH exhibited enrichment of pro-metastatic components activated Notch1, JAG1/2; MMPs; and ADAMs.(P) Representative protein cargoes of exosomes and ectosomes released by MDA-MB-231 cells expressing empty vector as deciphered with proteomics using Mass Spectrometry. *p < 0.05; **p < 0.01; ***p < 0.001. [file 12943_2019_1077_MOESM4_ESM.jpg]

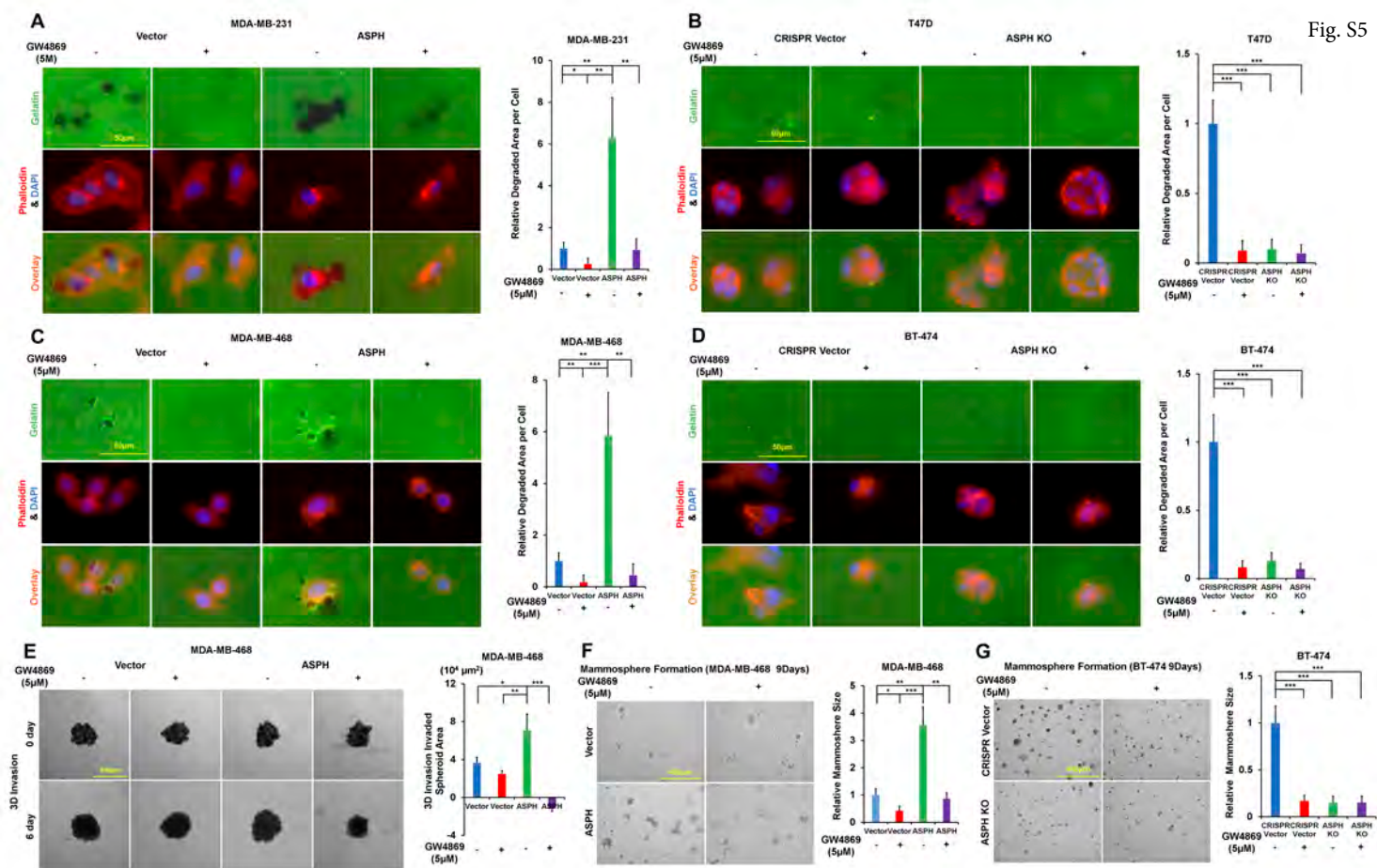

Supplement: Supplementary file 5 — Additional file 5: Figure S5. Inhibition of exosomes synthesis/release efficiently attenuated ASPH-mediated malignant phenotypes of breast cancer cells. (A-D) ECM degradation/remodeling in MDA-MB-231, T47D, MDA-MB-468 or BT474 cells, respectively, in response to GW4869. (E) 3-D invasion of MDA-MB-468 cells in response to GW4869. (F-G) Mammosphere formation of MDA-MB-468 or BT47 cells in response to GW4869. *p < 0.05; **p < 0.01; ***p < 0.001. [file 12943_2019_1077_MOESM5_ESM.pdf]

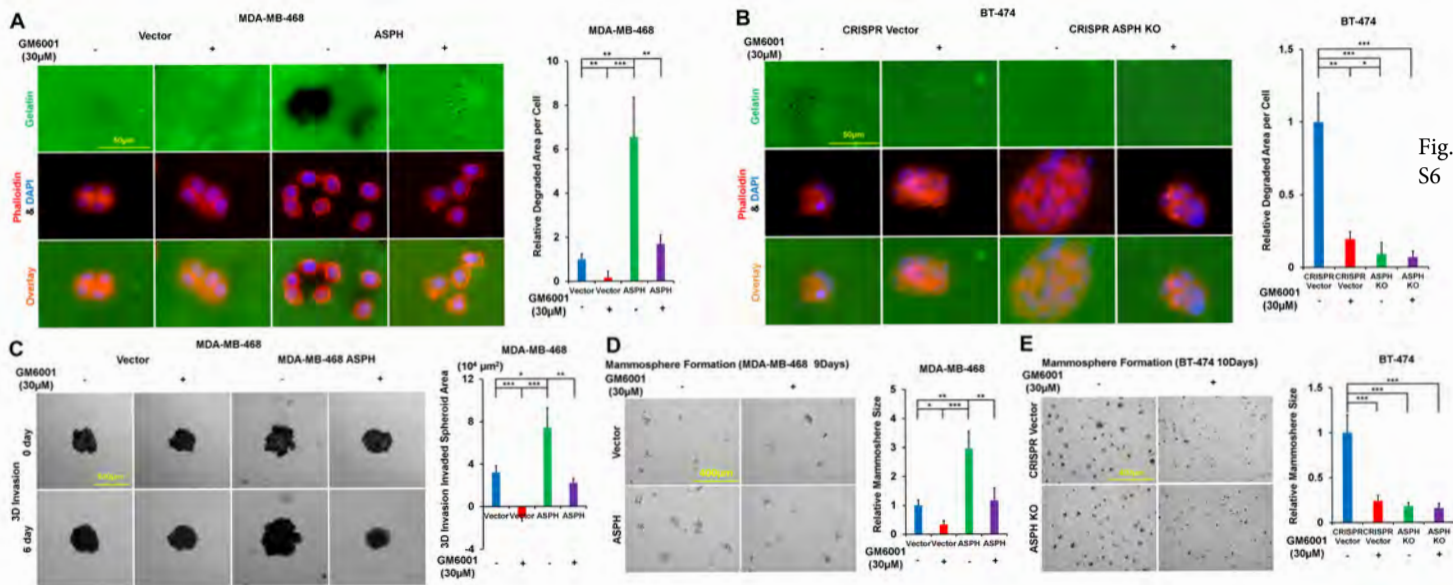

Supplement: Supplementary file 6 — Additional file 6: Figure S6. Inhibition of MMPs activity dramatically reduced ASPH rendered cellular behaviors in breast cancer. (A-B) ECM degradation/remodeling in MDA-MB-468 or BT474 cells in response to GM6001. (C) 3-D invasion of MDA-MB-468 cells in response to GM6001. (D-E) Mammosphere formation of MDA-MB-468 or BT47 cells in response to GM6001. *p < 0.05; **p < 0.01; ***p < 0.001. [file 12943_2019_1077_MOESM6_ESM.pdf]
