# Supplementary material for: ASPH-notch Axis guided Exosomal delivery of Prometastatic Secretome renders breast Cancer multi-organ metastasis
Source: Mol Cancer. 2019 Nov 7;18:156. doi: 10.1186/s12943-019-1077-0 (PMC6836474; doi:10.1186/s12943-019-1077-0)
Supplement: Supplementary file 7 — Additional file 7: Table S1. Demographic and clinical characteristics of breast cancer patients (validation set). [file 12943_2019_1077_MOESM7_ESM.docx]

Supplemental Table 1. Demographic and clinical characteristics of breast cancer patients (validation set)

| Variables | No. of patients (%) |
| --- | --- |
| Age (years) | |
| ≤ 35 | 16 (11.3) |
| > 35 | 125 (88.7) |
| TNM stage | |
| 0 | 9 (6.4) |
| I | 75 (53.2) |
| II | 44 (31.2) |
| III | 13 (9.2) |
| Surgery |  |
| Mastectomy | 140 (99.3) |
| BCS | 1 (0.7) |
| Tumor size (cm) | |
| < 2 | 58 (41.1) |
| 2-5 | 80 (56.7) |
| > 5 | 3 (2.1) |
| Lymph node involvement (n) | |
| 0 | 73 (54.5) |
| 1-3 | 27 (20.1) |
| 4-9 | 21 (15.7) |
| ≥10 | 13 (9.7) |
| Histological grade | |
| I-II (Well-Moderate differentiation) | 21 (14.9) |
| III-IV (Poor-Dedifferentiation) | 120 (85.1) |
| ER status |  |
| (+) | 96 (68.1) |
| (-) | 45 (31.9) |
| PR status |  |
| (+) | 87 (61.7) |
| (-) | 54 (38.3) |
| HER2 status | |
| (+) | 72 (51.1) |
| (-) | 69 (48.9) |
| Ki-67 index (%) | |
| ≤ 14 | 56 (39.7) |
| > 14 | 85 (60.3) |
| P53 status | |
| (+) | 38 (35.8) |
| (-) | 68 (64.2) |
| Pathology type |  |
| Paget’s disease | 2 (1.4) |
| DCIS | 10 (7.1) |
| IDC | 121 (85.8) |
| ILC | 6 (4.3) |
| Phyllodes tumor | 1 (0.7) |
| Neuroendocrine tumor | 1 (0.7) |
| Molecular subtype | |
| Luminal A | 23 (19.2) |
| Luminal B | 57 (47.5) |
| TNBC | 15 (12.5) |
| HER2 amplified | 25 (20.8) |
| Recurrence/Metastasis | |
| No | 119 (84.4) |
| Yes | 22 (15.6) |
| Chemotherapy | |
| No | 21 (24.1) |
| Yes | 66 (75.9) |

BCS, breast-conserving surgery; DCIS, ductal carcinoma in situ, including comedocarcinoma in situ (1 case); IDC, invasive ductal carcinoma including Mucinous (7) and Papilloma (1) subtypes; ILC, invasive lobular carcinoma.

|  |  |  |  |
| --- | --- | --- | --- |
